# Weight spectrum and executive function in adolescents: the moderating role of negative emotions

**DOI:** 10.1186/s13034-022-00468-9

**Published:** 2022-05-09

**Authors:** Qingmin Lin, Yanrui Jiang, Xiaoning Sun, Yunting Zhang, Wenjie Shan, Jin Zhao, Xuelai Wang, Qi Zhu, Wanqi Sun, Hui Lu, Fan Jiang

**Affiliations:** 1 School of Life Science and Biotechnology, Shanghai Jiao Tong University, Shanghai, 200240 China; 2 Department of Developmental and Behavioral Pediatrics, Pediatric Translational Medicine Institution, Shanghai Children’s Medical Center, School of Medicine, Shanghai Jiao Tong University, 1678 Dongfang Rd., Shanghai, 200127 China; 3 Ministry of Education-Shanghai Key Laboratory of Children’s Environmental Health, Xinhua Hospital, School of Medicine, Shanghai Jiao Tong University, Shanghai, 200092 China; 4 Child Health Advocacy Institute, Shanghai Children’s Medical Center, School of Medicine, Shanghai Jiao Tong University, Shanghai, 200127 China; 5 Department of VIP Clinic, Shanghai Children’s Medical Center, School of Medicine, Shanghai Jiao Tong University, Shanghai, 200127 China; 6 Shanghai Mental Health Center, School of Medicine, Shanghai Jiao Tong University, Shanghai, 200030 China; 7 Shanghai Center for Brain Science and Brain-Inspired Technology, Shanghai, 201602 China

**Keywords:** Adolescents, Weight spectrum, Abdominal weight, Executive function, Depression

## Abstract

**Background:**

While recent works suggested that overweight/obesity may impair executive function (EF), the overweight/obesity-EF relationship has not been well studied in adolescents. Furthermore, no research has investigated adolescent EF impairments across the weight spectrum (e.g., underweight or thinness, normal, overweight/obesity), especially those with underweight condition, with the moderating effect of negative emotions in the weight-EF association being limitedly investigated. We aimed to determine whether overall and abdominal weight spectrum associated with EF impairments and to identity whether negative emotions moderate the weight-EF link in adolescents.

**Methods:**

We applied a subsample of the SCHEDULE-A project. Adolescents (11–18 years) were recruited using a multi-stage cluster random sampling approach. We measured the overall and abdominal weight spectrum by body mass index z-score and waist-to-height ratio, respectively. We used the Behavior Rating Inventory of Executive Function (BRIEF) to evaluate adolescent EF in nature setting, and utilized the Depression Anxiety and Stress Scales (DASS-21) to assess three types of negative emotional status (i.e., depression, anxiety, and stress).

**Results:**

Of the 1935 adolescents, 963 (49.8%) were male. We observed that abdominal, not overall, overweight was associated with the Global Executive Composite (GEC) impairment (OR = 1.59, 95% CI 1.07–2.35), particularly for inhibit, emotion control, shift, working memory, and monitor domains. Furthermore, depression moderated the abdominal overweight-GEC association (P = 0.032 for interaction term), especially for emotional control, working memory, and initiate dimensions. Moreover, we also found abdominal thinness was associated with the Metacognition Index problem (OR = 1.33, 95% CI 1.04–1.72), particularly for plan and monitor areas.

**Conclusions:**

Both abdominal overweight and thinness were associated with adolescent EF, and depression would be a modifiable target to improve EF in adolescents with abdominal overweight. Future longitudinal studies are needed to investigate the causal relationship between abdominal weight spectrum and EF, as well as the underlying mechanisms among adolescents suffering from depression.

**Supplementary Information:**

The online version contains supplementary material available at 10.1186/s13034-022-00468-9.

## Background

Nearly 50 years, overweight/obesity has become a global pandemic, especially in children with a much larger rate of increase [1]. While the increasing trend of overweight/obesity prevalence has flattened in developed western countries, such increase, however, is still accelerating in most developing Asian countries [2], with China having the largest number of children and adolescents with excessive body weight [3]. It is worth noting that children’s overweight/obesity, especially in adolescence, is more likely to persist into adulthood, not only predisposing individuals to a bulk of adverse physical consequences such as metabolic syndrome, cardiovascular disease, type 2 diabetes, and cancers [1, 4], but also leading to a high risk of lower academic achievement and cognitive impairment [5].

Executive function (EF), a sub-domain of cognitive capacity, also called cognitive or executive control, refers to a set of top-down neurocognitive mental processes against one’s bottom-up instinct actions (including three core subcomponents: inhibitory control, working memory, and cognitive flexibility), which is necessary to make one’s decisions and engage in one’s purposeful and goal-driven behaviors [6]. While recent works suggested that overweight/obesity may increase the risk of EF issues [7, 8], the overweight/obesity-EF relationship in adolescents has not been well studied [9, 10]. Specifically, some studies reported adolescents with overweight/obesity, or higher body mass index (BMI) value, had poor EF performance [11, 12], while others showed null results [13, 14]. For instance, one recent study reported that not overweight/obesity per se exerted negative impact on EF performance [14]. Importantly, few research has investigated EF impairments across weight spectrum (e.g., underweight, normal, overweight/obesity) [15] in adolescents, especially those with underweight condition. In the meantime, most studies had a limited sample size (often by case–control study design), only used the BMI indicator (less sensitive to body fat [16]) to assess overweight/obesity condition, and often neglected the group with underweight state that likely present with a lower EF score [17]. These limitations likely lead to the above-mentioned equivocal findings, hence hindering a comprehensive understanding of the weight-EF link.

More importantly, one possible reason for the aforementioned conflicting findings is that the weight-EF association is likely contingent on some factors. In other words, weight may relate to EF only among certain adolescents. Negative emotions (e.g., depression) frequently occur in adolescence, a period known for significant changes in the body, the brain and the mind, as well as the social environment around them [18]. Adolescents with weight concerns may tend to experience negative self-image and more maladaptive behaviors (e.g., unhealthy eating, physical inactivity, and more screen exposure) and, in turn EF problems [19], alternatively, negative emotions may serve to exacerbate the weight issue [20], which gave us a hint that negative emotions might moderate the weight-EF relationship. Moreover, one adult research has shown that the EF difference between obesity and normal weight group was more prominent in patients with major depressive disorder compared to healthy controls [21]. However, much of the current literature only focused on the solitary effect of weight condition on EF in adolescents [11–14], making it difficult to determine the extent to which the weight-EF association may be contingent on negative emotions. Given that a growing number of adolescents suffer from weight problems, it is crucial to identify modifiable targets for the EF improvement.

To address these gaps, the current study aimed to determine whether overall and abdominal weight spectrum (including underweight, normal, and overweight/obesity) significantly associated with EF impairment and to identity whether negative emotions (e.g., depression, anxiety, and stress) moderate the weight-EF association in adolescents. We hypothesized that both overweight/obesity and underweight condition would significantly associate with EF impairment in adolescents, especially abdominal non-normal weight state. We also hypothesized that all three types of negative emotions would significantly moderate the weight-EF association. That is, overweight and underweight condition would significantly associated with EF problem only among those experiencing negative emotions.

## Methods

### Study sample

From the study of the Shanghai Children’s Health, Education and Lifestyle Evaluation-Adolescents (SCHEDULE-A), a population-based cross-sectional survey investigating adolescent physical and mental health (11–18 years), the current study utilized a subsample of adolescents recruited from Shangrao region (December 2018), a relatively social-economic underdeveloped city located in the downstream Yangtze River in southeast China. The subsample included both overall and abdominal weight indicators, and also had a relatively high underweight rate that allowed us to examine the underweight-EF relationship. A multi-stage cluster random sampling approach (i.e., District-School-Class-Student) was used. Details were reported in Additional file 1: Method S. Briefly, four districts/counties were firstly sampled according to the per capita disposal income of the Chinese residents in 2016; next, in each sampled district/county, two junior and two senior high schools were selected at random (4 districts/counties × 4 schools); then one class from each grade of the selected schools was drawn randomly; finally, all students were invited to take part in the study. The protocol was approved by the Shanghai Children's Medical Center Human Ethics Committee according to the Declaration of Helsinki (SCMCIRB-K2018103). All parents and their adolescent children provided written informed consents.

**Fig. 1:**
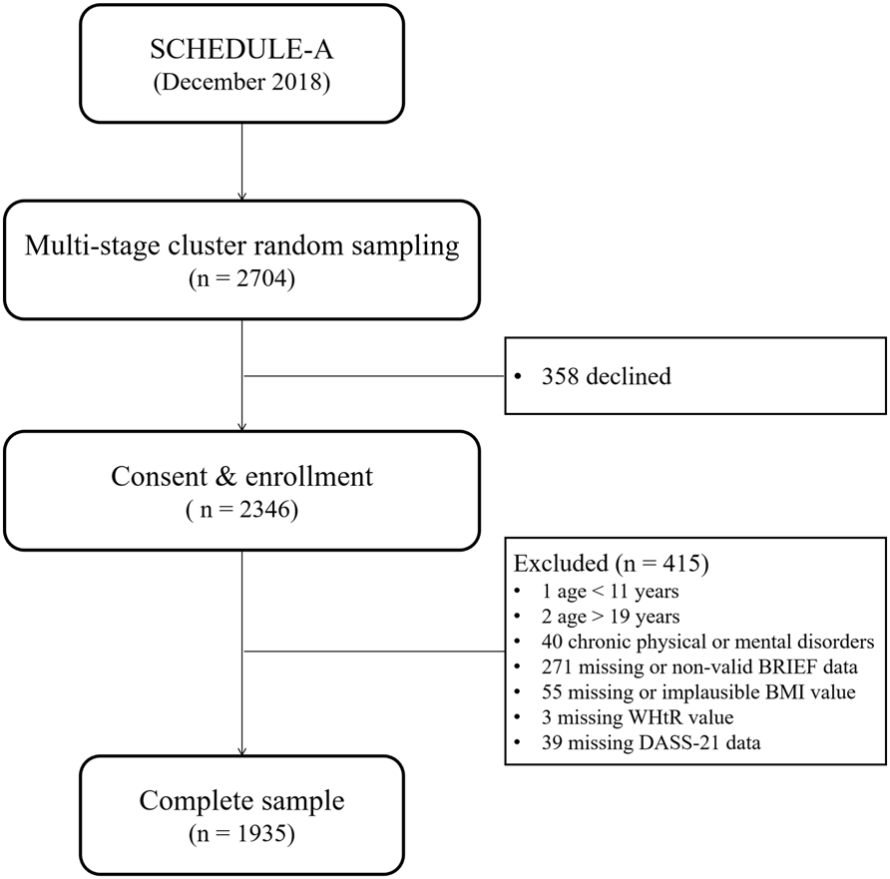
Flowchart of the participants. *BMI* body mass index, *BRIEF* the Behavior Rating Inventory of Executive Function, *DASS* the Depression Anxiety and Stress Scales with 21 items, *SCHEDULE-A* study of the Shanghai Children’s Health, Education and Lifestyle Evaluation-Adolescents, *WHtR* waist-to-height ratio

Through the above-mentioned sampling method, 2704 students were selected, and 2346 students (86.8% response rate) agreed to participate. Finally, a total of 1935 students were retained after data cleaning based on the following inclusion criteria: (1) age ranged from 11 to 18 years; (2) without any chronic physical and mental disorders; and (3) without missing and invalid data of the main variables, i.e., weight, negative emotions, and EF (Fig. 1).

### Main variables

#### Weight spectrum

We obtained information on adolescents’ height, weight, and waist circumstance from each school, which were measured by staffs of the school infirmary. BMI was calculated as the weight divided by the squared height, and waist-to-height ratio (WHtR) was computed as the waist circumference divided by the height. BMI value was subsequently converted into z-score using the least mean square method according to the World Health Organization growth reference [22]. Then, we defined BMI z-score <  − 1,  − 1 ~ 1, ≥ 1, and ≥ 2 as the overall weight spectrum, i.e., underweight, normal, overweight, and obesity, and defined WHtR < 0.40, 0.40 ~ 0.46, ≥ 0.46, and ≥ 0.50 as the abdominal weight spectrum, including thinness, normal, overweight, and obesity, respectively [23, 24].

#### Negative emotions

We assessed three types of adolescent negative emotional status (i.e., depression, anxiety, and stress) by self-report, using the Chinese version of the Depression Anxiety and Stress Scales with 21 items (DASS-21). This version was validated in mainland China with internal consistency indices (Cronbach's alpha) of 0.83, 0.80, and 0.82, and test–retest reliability of 0.39, 0.43, and 0.46 for the depression, anxiety, stress domains, respectively, which supported its potential clinical utility in Chinese population [25]. All items were rated on a four-point Likert-type scale from 0 “did not apply to me at all” to 3 “applied to me very much or most of the time”. Each domain includes seven items. Specifically, item 3, 5, 10, 13, 16, 17, and 21 belong to depression domain, item 2, 4, 7, 9, 15, 19, and 20 belong to anxiety domain, and item 1, 6, 8, 11, 12, 14, and 18 belong to stress domain. The summed score for each domain was multiplied by 2, and higher scores indicated greater negative emotional symptoms. Adolescent who scored moderate or above with the cutoffs of ≥ 14, ≥ 10, and ≥ 19 were classified as experiencing depression, anxiety, and stress issues, respectively.

#### Executive function

We measured adolescent EF in a natural setting by parent report, using the Chinese version of the Behavior Rating Inventory of Executive Function (BRIEF), which can serve as a screening tool for possible executive dysfunction in children and adolescents aged 5–18 years old [26]. The BRIEF has eight non-overlapping domains, and almost all showed a good internal consistency (0.74–0.96) and test–retest reliability (0.68–0.89) in the Chinese population [27]. Of the total 86 items, each one is rated on a three-point scale (i.e., never, sometimes and often), and parents were required to select the most suitable answer for each described behavior of their adolescents in the past 6 months. For a few individuals aged over 18 years (n = 137), we utilized the same version, as they lived together with their parents, and their everyday function therefore could be well-assessed by their parents [28]. We checked the raw data based on two validity indexes (i.e., negativity < 5 and inconsistency < 7) to reduce reporting bias according to the BRIEF manual.

Eight domains of the BRIEF can form two broader indices and one overall index. The broad Behavior Regulation Index (BRI) includes three domains, i.e., inhibit, emotional control, and shift, which are interpreted as the ability to regulate one’s own behavioral and emotional control, and to move flexibly from one action to another. The broad Metacognition Index (MI) incorporates five domains, i.e., working memory, plan, initiate, organize, and monitor, which are related to the ability to solve problem actively, and to initiate, organize, and monitor one’s own actions. All eight domains form the overall index, i.e., the Global Executive Composite (GEC). T-scores were computed based on sex- and age-specific norms, with the T-score > 60 and > 65 being defined as potential sub-clinical and clinical EF impairments or problems.

### Confounders

According to a recently suggested principle of confounder selection, we selected several covariates that may influence the association between weight spectrum and EF, such as social-demographic factors (i.e. adolescents’ age, sex, parental highest education, and family gross income) and individual lifestyle behaviors (i.e. screen exposure, nighttime sleep duration, and physical activity) [29]. Parents or other main caregivers reported the socio-demographic information, including parents’ educational attainment, gross household income, and adolescents’ sex, birth date, and some chronic physical and mental health status. Screen exposure was measured by two widely used questions in children and adolescents [30], that is in the last month, on average, the total time he/she spent per day on (1) sitting and watching television or videos, and (2) playing games using device such as cellphone, iPad, PlayStation, etc. Each response was dichotomized, with ≥ 2 h/day indicating excessive screen time. Adolescents were also asked “At what time do you usually go to bed and get up on weekdays and weekends, respectively?” Averaged night sleep duration (i.e., time in bed in current study) was calculated by (5*weekdays + 2*weekends)/7, then was classified as shorter duration by cutoffs of < 9, < 8, and < 7 h for adolescents aged 12–13, 14–17, and ≥ 18 years, respectively [31]. We used a short Chinese version of the International Physical Activity Questionnaire to measure adolescent physical activity intensity, and then categorized it into low, moderate and high level [32].

### Statistical analysis

Participants’ characteristics were described with mean (SD) and frequency (%), and the social-demographic differences between analyzed and excluded sample were assessed by t-test and chi-squared test for continuous and categorical variables, respectively.

In considering of a non-linear association between weight and EF, we fitted a linear regression model with BMI z-score and WHtR included as both a linear and quadratic term for each EF domain. Because almost all BMI z-score quadratic terms were not statistically significant (Additional file 1: Fig. S1), but WHtR quadratic terms were (Additional file 1: Fig. S2), two overall weight categories (i.e., normal and overweight), and three abdominal weight categories (i.e., thinness, normal, and overweight) were used in the subsequent analyses (because of a low obesity prevalence, these adolescents therefore were included in the overweight category, Table 1).

**Table 1:** Participant characteristics

Characteristic	Participants, mean ± SD / No. (%)
Age, y	15.32 ± 1.79
Sex	
Boys	963 (49.8)
Girls	972 (50.2)
Household factors	
Parental highest education	
Lower than high school	1374 (73.2)
High school or higher	503 (26.8)
Family income (RMB)	
< 50,000	939 (58.7)
≥ 50,000	661 (41.3)
Individual behaviors	
Screen exposure time	
Sitting&Watching, ≥ 2 h/day	868 (44.9)
Playing games, ≥ 2 h/day	769 (39.8)
Sleep duration, short	978 (51.1)
Physical activity	
Low	557 (28.8)
Moderate	710 (36.7)
High	666 (34.5)
Predictors	
Overall weight spectrum (BMI z-score)	
Underweight, < -1	436 (22.5)
Overweight, ≥ 1	215 (11.1)
Obesity, ≥ 2	32 (1.7)
Abdominal weight spectrum (WHtR)	
Thinness, < 0.40	708 (36.6)
Overweight, ≥ 0.46	442 (22.8)
Obesity, ≥ 0.50	188 (9.7)
Potential Moderators	
Depression, ≥ 14	492 (25.4)
Anxiety, ≥ 10	1022 (52.8)
Stress, ≥ 19	379 (19.6)
Executive function problems	
GEC score	
Sub-clinical problem, > 60	393 (20.3)
Clinical problem, > 65	176 (9.1)
BRI score	
Sub-clinical problem, > 60	340 (17.6)
Clinical problem, > 65	224 (11.6)
MI score	
Sub-clinical problem, > 60	399 (20.6)
Clinical problem, > 65	127 (6.6)

*BMI* body mass index, *BRI* the Behavioral Regulation Index, *GEC* the Global Executive Composite, *MI* the Metacognition Index, *WHtR* waist-to-height ratio

We used multivariable logistic regression with standard error type of clustered robust to determine the association of weight spectrum and negative emotion with each sub-clinical EF problem (because of a low prevalence of clinical EF issues, adolescents with clinical EF issues, therefore, were included in the sub-clinical category, Table 1). The potential moderating effect of each negative emotion on the weight-EF link was tested by adding an interactive term (e.g., depression × overweight) in the model. Should the interaction term reached statistical significance, simple effect analysis was subsequently performed. We further conducted a post-hoc analysis of the associations between negative emotions and weight spectrum using multinomial logistic analysis. Finally, we performed a sensitivity analysis by limiting adolescents with WHtR values within 0.2–0.7 (removing possible outliers, Additional file 1: Fig. S2), and by omitting individuals with age over 18 years (the BRIEF was developed for children and adolescents aged 5–18 years).

All analyses were performed in Stata 15.0, and P values < 0.05 were considered statistical significance.

## Results

### Descriptive characteristics

Of the final 1935 adolescents (Fig. 1), the mean age was 15.32 ± 1.79 years and 963 (49.8%) were male (Table 1). 26.8% of adolescents’ parents obtained a high school education or more, and 41.3% of their family income were ≥ 50,000 RMB. Moreover, approximately 40% participants had each type of screen exposure exceeding 2 h/day, 50% were shorter sleepers, and 30% displayed low physical activity. There were no significant social-demographic differences between the analyzed and excluded individuals (Additional file 1: Table S1).

### Association of weight spectrum and EF problems

After adjusting for confounders, we observed that abdominal, not overall, overweight had a significant association with GEC problem (OR = 1.59, 95% CI 1.07–2.35). However, we didn’t find abdominal thinness significantly associated with GEC issue (Table 2).

**Table 2:** Association of overall and abdominal weight spectrum, as well as negative emotions with executive function problems in adolescents

	GEC problem		BRI problem		MI problem	
	OR (95% CI)	P value	OR (95% CI)	P value	OR (95% CI)	P value
Model a–e^a^						
Overall weight status						
Normal	Ref		Ref		Ref	
Overweight	1.01 (0.65, 1.58)	0.949	0.92 (0.53, 1.61)	0.782	1.17 (0.63, 2.17)	0.613
Abdominal weight spectrum						
Normal	Ref		Ref		Ref	
Thinness	1.31 (0.88, 1.95)	0.179	1.19 (0.57, 2.48)	0.645	**1.33 (1.04, 1.72)**	**0.025**
Overweight	**1.59 (1.07, 2.35)**	**0.021**	1.59 (0.93, 2.70)	0.089	**1.55 (1.21, 1.98)**	** < 0.001**
Depression						
No	Ref		Ref		Ref	
Yes	**3.38 (1.35, 8.44)**	**0.009**	**2.95 (1.08, 8.09)**	**0.036**	**3.03 (1.45, 6.36)**	**0.003**
Anxiety						
No	Ref		Ref		Ref	
Yes	**2.78 (1.65, 4.70)**	** < 0.001**	**3.04 (1.85, 4.99)**	** < 0.001**	**2.44 (1.36, 4.39)**	**0.003**
Stress						
No	Ref		Ref		Ref	
Yes	**1.75 (1.07, 2.87)**	**0.026**	**1.44 (1.04, 1.97)**	**0.026**	1.48 (0.96, 2.29)	0.076

The bold words represent the P values less than 0.05

*BRI* the Behavioral Regulation Index, *GEC*, the Global Executive Composite, *MI*, the Metacognition Index

^a^All models were adjusted for social-demographic factors (i.e. age, sex, parental highest education, and family gross income) and individual lifestyle behaviors (i.e. screen exposure, nighttime sleep duration, and physical activity)

In sub-domain analyses (Table 2, Additional file 1: Table S2), the abdominal overweight significantly associated with MI problem (OR = 1.55, 95% CI 1.21–1.98), especially in working memory and monitor. Furthermore, despite of a borderline association between abdominal overweight and BRI issue (OR = 1.59, 95% CI 0.93–2.70), all three domains (i.e., inhibit, emotion control, and shift) reached statistical significance. Moreover, we also found that abdominal thinness obviously associated with MI problem (OR = 1.33, 95% CI 1.04–1.72), especially in plan and monitor.

### Moderating effect of negative emotions in the weight-EF link

After adjusting for confounders, the negative emotions, especially depression and anxiety symptoms were strongly associated with adolescents GEC problem, with ORs being 3.38 (95% CI 1.35–8.44), 2.78 (95% CI 1.65–4.70), and 1.75 (95% CI 1.07–2.87) for depression, anxiety, and stress, respectively (Table 2).

**Fig. 2:**
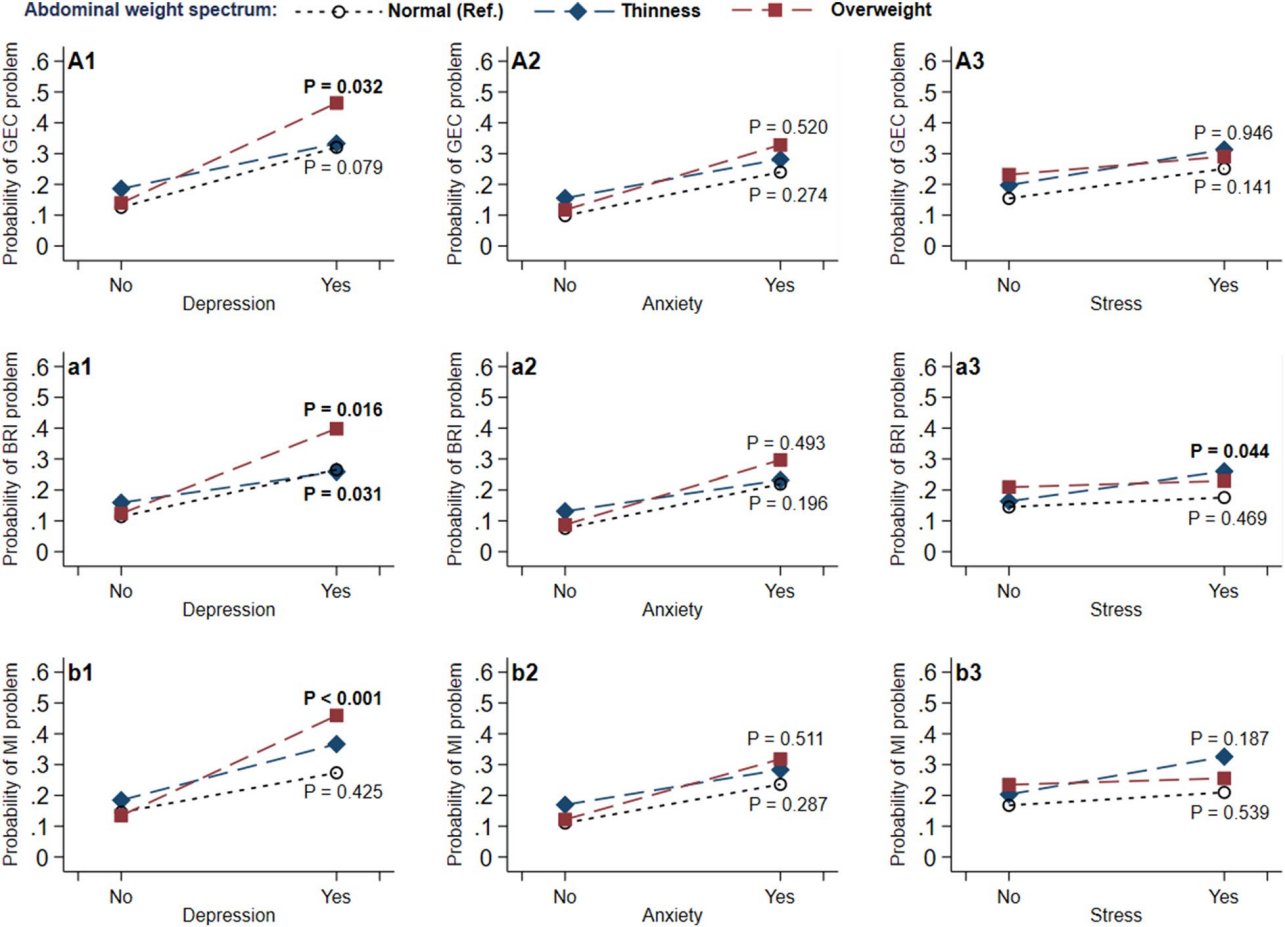
Moderating effects of negative emotions on the association between abdominal weight spectrum and executive function problems in adolescents. All models were adjusted for sociodemographic characteristics (i.e., age, sex, parental education level, and gross family income) and individual behaviors (screen time, night sleep duration, and physical activity), and the P values indicated whether each interaction term of negative emotions (i.e., depression, anxiety, and stress) and abdominal weight spectrum (i.e., abdominal thinness, and overweight) on executive dysfunction reached statistical significance. *BRI* the Behavioral Regulation Index, *GEC* the Global Executive Composite, *MI* the Metacognition Index

In the interactive (Additional file 1: Table S3, Fig. 2A1–A3 and simple (Table 3) effect analyses, we only found depression positively moderated the association of abdominal overweight and GEC problem (P = 0.032). That is in adolescents who suffered from depression, abdominal overweight significantly associated with GEC impairment (OR = 1.92, 95% CI 1.26–2.93); whereas, in individuals free from that symptoms, no significant effect was found. Additionally, we also observed a borderline negative moderating effect of depression on the link of abdominal thinness and GEC problem (P = 0.08), namely in individuals without depression, abdominal thinness significantly associated with GEC impairment (OR = 1.51, 95% CI 1.10–2.08).

**Table 3:** Simple effects of abdominal weight spectrum on executive function problems stratified by depression condition in adolescents^a^

	GEC problem		BRI problem		MI problem	
	OR (95% CI)	P value	OR (95% CI)	P value	OR (95% CI)	P value
No depression						
Abdominal weight spectrum						
Normal	Ref		Ref		Ref	
Thinness	**1.51 (1.10, 2.08)**	**0.010**	1.37 (0.67, 2.80)	0.386	**1.30 (1.01, 1.66)**	**0.042**
Overweight	1.14 (0.80, 1.63)	0.472	1.09 (0.73, 1.64)	0.666	0.96 (0.72, 1.26)	0.757
Depression						
Abdominal weight spectrum						
Normal	Ref		Ref		Ref	
Thinness	1.01 (0.52, 1.95)	0.974	0.94 (0.41, 2.15)	0.887	1.46 (0.90, 2.36)	0.122
Overweight	**1.92 (1.26, 2.93)**	**0.003**	**2.17 (1.30, 3.63)**	**0.003**	**2.44 (1.82, 3.26)**	** < 0.001**

The bold words represent the P values less than 0.05

*BRI* the Behavioral Regulation Index, *GEC* the Global Executive Composite, *MI* the Metacognition Index

^a^Adjusted for social-demographic factors (i.e. age, sex, parental highest education, and family gross income) and individual lifestyle behaviors (i.e. screen exposure, nighttime sleep duration, and physical activity)

In sub-domain interactive (Additional file 1: Table S3–S4, Fig. 2, Additional file 1 Fig. S3) and simple (Table 3, Additional file 1: Table S5) effect analyses, similar findings were observed that depression positively moderated the link of abdominal overweight with BRI (P = 0.016) and MI (P < 0.001) problems, especially for emotional control (borderline), working memory, and initiate. That is abdominal overweight significantly associated with BRI (i.e., emotion control) and MI (i.e., working memory, and initiate) impairments only in adolescents who endorsed depression. While we observed a significant negative moderating effect of depression on the link of abdominal thinness and BRI problem (P = 0.031), the simple effect analysis did not reveal significant findings.

Although we observed that stress positively moderated the link of abdominal thinness and BRI issue (P = 0.044) (Additional file 1: Table S3 and Fig. 2a3), it didn’t reach statistical significance of the moderating effect in any specific sub-domains (Additional file 1: Fig. S4). We also didn’t find anxiety had significant moderating effect. The post-hoc analysis also showed that only depression significantly associated with abdominal overweight (Additional file 1: Table S7).

### Sensitivity analysis

After limiting adolescents’ WHtR value and age range, the results did not change much indicating the robustness of our findings (Additional file 1: Table S7–S12, Additional file 1: Table S13–S18).

## Discussion

To our knowledge, this is the first study to examine whether negative emotions moderate the weight-EF link in adolescents, taking into consideration both overall and abdominal weight spectrum. There are several important findings. Firstly, we observed abdominal overweight, rather than overall overweight, significantly associated with EF problem. Furthermore, we identified a statistically significant moderating effect of depression in the relationship of abdominal overweight and EF issue. Moreover, we also found abdominal thinness significantly associated with EF impairment.

We observed a significant association of abdominal, not overall, overweight with EF problems, particularly in the areas of inhibit, emotion control, shift, working memory, and monitor. This finding was consistent with two prior findings indicating that waist circumstance or WHtR was more sensitive than BMI indicator when examining the weight-EF association [33, 34]. Consistent with our findings, prior studies also suggested overweight had pronounced effect on the deficits of inhibit control, working memory, cognitive flexibility (i.e., shift in our study), as well as high-order EF (e.g., reason, problem-solve, and plan) [9]. The underlying mechanism of the overweight-EF link may be attributed to chronic low-grade inflammation. Extensive studies have documented that overweight, especially abdominal adiposity (a marker of visceral fat), can induce systemic low-grade inflammation through activating pro-inflammatory processes in adipose tissue by secreting chemokines and in gut microbiota by altering intestinal permeability and releasing endotoxins [35, 36]. Inflammation can result in decreased brain volume, reduced white matter integrity, atrophied grey matter, and lower regional blood flow, all of which likely damage EF if occurred in EF-related regions, e.g., frontal, temporal and occipital cortices [7].

Furthermore, we also found depression significantly moderated the link of abdominal overweight and EF impairment in adolescents, especially in the domains of emotional control, working memory, and initiate. Our finding corroborates with one adult research showing that obesity condition interacts with major depressive disorder to influence EF performance [21]. Research showed that individuals who suffered from overweight were more vulnerable to depression due to body dissatisfaction and peer stigma than those who had normal weight [37]. Meanwhile, persons with depressed symptoms were more likely to gain weight and had poor metabolic profile given increased unhealthy diet, sleep disorder, and less physical activity [38]. A recent longitudinal study also demonstrated that higher weight predicted more severe depressive symptoms and worse EF via higher levels of IL-6, and depressive symptoms also predicted IL-6 increase, and all observed associations were unique to depression but not anxious [39]. Adolescence is widely recognized as a stage of heightened emotional reactions [18], and depressive status measured by DASS-21 in our study likely predispose these adolescents to clinical disorder if not properly addressed, hence warranting much more attention.

Moreover, we observed a quadratic relation between WHtR and EF profile, similar to one recent finding showing a U-shape relationship of BMI with brain volume in children [17]. Besides aforementioned abdominal overweight, we also found abdominal thinness significantly associated with EF impairment in adolescents, with, however, other studies reporting null findings, possibly due to the BMI indicator being used [13]. Because of undernutrition in low income region, or self-pressure to restrict dieting fulfilling the thin beauty ideal [40] (oftentimes by unhealthy eating [41]), individuals with abnormal thinness/underweight probably have lower than desired levels of nutritional and biochemical molecule for optimal EF, e.g., protein, micronutrient, IGF-1 and brain derived neurotrophic factor [42, 43]. Future studies using more sensitive indicators such as body composition and biochemical markers are needed to confirm and extend our finding.

Our study neither found a significant association of overall overweight and EF, nor observed a moderating effect of depression on that association. It is possible that the BMI indicator incorporating both lean and fat mass is an insufficient marker of body fat. Although one adult study found stress significantly moderated the abdominal obesity and EF association [44], our study didn’t find stress and anxiety as statistically significant moderators. Our post-hoc analysis indicating that anxiety and stress were not associated with abdominal overweight may be one of the reasons. More mechanism researches of the association between different types of negative emotions and abdominal overweight in adolescents should be conducted in the future.

### Limitations

The present study had some limitations. Firstly, our sample was from one of the underdeveloped cities in China with a relatively low obesity prevalence. More samples with diverse socioeconomic status should be performed in future. Secondly, due to low rate of obesity and clinical EF impairment, we utilized the overweight and sub-clinical EF cutoffs. Although the effects estimated were at magnitudes that may be considered sub-clinical in adolescents, these findings are important at the population level. Thirdly, adolescent lifestyle behaviors relied on self-report. Future studies should use more objective measurements, e.g., using actiwatch to assess night sleep duration and physical activity, to reduce reporting bias. Fourthly, we did not collect the adolescents’ perspectives on their EF which will influence our interpretation of the findings. Fifthly, that we performed lots of regressions may increase the type I errors, which should be taken into consideration when interpreting the findings. And lastly, given the cross-sectional nature, we cannot determine the causal association, and cannot exclude the effects influenced by other unmeasured or unknown confounders.

## Conclusions

Our study confirmed and extended previous researches by examining the association of overall and abdominal weight spectrum with EF, and determining the moderating effect of negative emotions on the weight-EF link in adolescents. Our findings highlighted that in future adolescent health actions, the WHtR was a more sensitive indicator of EF than BMI that needs more attention, and the probable depression was a modifiable target to improve EF in adolescents with abdominal overweight that requires more care. Future longitudinal studies are also needed to investigate the causal role of abdominal weight spectrum on EF, as well as its underlying mechanism among adolescents suffering from depression symptoms.

## Supplementary Information

Additional file 1: **Figure S1. **Scatter and quadratic fitted plots of BMI z-score and executive function scores in adolescents. **Figure S2.** Scatter and quadratic fitted plots of WHtR and executive function scores in adolescents. **Figure S3**. Moderating effects of depression condition in the association between abdominal weight spectrum and executive function problems in adolescents. **Figure S4**. Moderating effects of stress condition in the association between abdominal weight spectrum and executive function problems in adolescents.** Table S1.** Participant characteristics between analyzed and excluded sample.** Table S2.** Association of overall and abdominal weight spectrum, as well as negative emotions with executive function problems in adolescents^1^.** Table S3.** Moderating effects of negative emotions in the association of overall and abdominal weight spectrum with executive function problems in adolescents^1^.** Table S4.** Moderating effects of depression in the association between abdominal weight spectrum and executive function problems in adolescents^1^. **Table S5. **Simple effects of abdominal weight spectrum on executive function problems stratified by depression condition in adolescents^1^.** Table S6.** Association of overall and abdominal weight spectrum, as well as negative emotions with executive function problems in adolescents. **Table S7.** Associations of negative emotions and abdominal weight spectrum in adolescents^1^.** Table S8. **Association of overall and abdominal weight spectrum, as well as negative emotions with executive function problems in adolescents^1^. **Table S9.** Moderating effects of negative emotions in the association of overall and abdominal weight spectrum with executive function problems in adolescents^1^.** Table S10.** Moderating effects of depression in the association between abdominal weight spectrum and executive function problems in adolescents^1^.** Table S11. **Simple effects of abdominal weight spectrum on executive function problems stratified by depression condition in adolescents^1^. **Table S12. **Simple effects of abdominal weight spectrum on executive function problems stratified by depression condition in adolescents^1^. **Table S13.** Association of overall and abdominal weight spectrum, as well as negative emotions with executive function problems in adolescents. **Table S14.** Association of overall and abdominal weight spectrum, as well as negative emotions with executive function problems in adolescents^1^.** Table S15.** Moderating effects of negative emotions in the association of overall and abdominal weight spectrum with executive function problems in adolescents^1^. **Table S16.** Moderating effects of depression in the association between abdominal weight spectrum and executive function problems in adolescents^1^. **Table S17. **Simple effects of abdominal weight spectrum on executive function problems stratified by depression condition in adolescents^1^. **Table S18. **Simple effects of abdominal weight spectrum on executive function problems stratified by depression condition in adolescents^1^.

## Data Availability

Data used for this study were derived from the SCHEDULE-A project. The datasets used and/or analyzed during the current study are available from the corresponding author on reasonable request.

## Abbreviations

BMI: Body mass index
BRI: The behavior regulation index
BRIEF: The Behavior Rating Inventory of Executive Function
DASS-21: The 21-item version of the Depression Anxiety and Stress Scales
EF: Executive function
GEC: The Global Executive Composite
MI: The metacognition index
SCHEDULE-A: Study of the Shanghai Children’s Health: Education and Lifestyle Evaluation-Adolescents
WHtR: Waist-to-height ratio
