# Supplementary material for: Weight spectrum and executive function in adolescents: the moderating role of negative emotions
Source: Child Adolesc Psychiatry Ment Health. 2022 May 9;16:34. doi: 10.1186/s13034-022-00468-9 (PMC9087912; doi:10.1186/s13034-022-00468-9)
Supplement: Supplementary file 1 — Additional file 1: Figure S1. Scatter and quadratic fitted plots of BMI z-score and executive function scores in adolescents. Figure S2. Scatter and quadratic fitted plots of WHtR and executive function scores in adolescents. Figure S3. Moderating effects of depression condition in the association between abdominal weight spectrum and executive function problems in adolescents. Figure S4. Moderating effects of stress condition in the association between abdominal weight spectrum and executive function problems in adolescents. Table S1. Participant characteristics between analyzed and excluded sample. Table S2. Association of overall and abdominal weight spectrum, as well as negative emotions with executive function problems in adolescents1. Table S3. Moderating effects of negative emotions in the association of overall and abdominal weight spectrum with executive function problems in adolescents1. Table S4. Moderating effects of depression in the association between abdominal weight spectrum and executive function problems in adolescents1. Table S5. Simple effects of abdominal weight spectrum on executive function problems stratified by depression condition in adolescents1. Table S6. Association of overall and abdominal weight spectrum, as well as negative emotions with executive function problems in adolescents. Table S7. Associations of negative emotions and abdominal weight spectrum in adolescents1. Table S8. Association of overall and abdominal weight spectrum, as well as negative emotions with executive function problems in adolescents1. Table S9. Moderating effects of negative emotions in the association of overall and abdominal weight spectrum with executive function problems in adolescents1. Table S10. Moderating effects of depression in the association between abdominal weight spectrum and executive function problems in adolescents1. Table S11. Simple effects of abdominal weight spectrum on executive function problems stratified by depression condition in a [file 13034_2022_468_MOESM1_ESM.docx]

**Method S**

Study of the Shanghai Children’s Health, Education and Lifestyle Evaluation-Adolescents (SCHEDULE-A), a population-based cross-sectional survey, was designed to investigate the individual, household and school-level factors of adolescent physical and mental health across regions with different social and economic development status. In SCHEDULE-A, one first-tier city (Shanghai, November 2017) and one third-tier city (Shangrao, December 2018) were chosen in order to include a sample that represented both developed and underdeveloped regions. The city tier distinctions were based on a classification system released by China Business News magazine in 2017, which was assessed according to five indicators: concentration of commercial resources, city’s pivotability, citizen vitality, variety of lifestyle, and flexibility in the future. Using a multi-stage cluster random sampling strategy, we recruited a representative school sample from the two selected cities. For the current study, we used data exclusively collected from Shangrao prefecture.

The multi-stage cluster sampling approach used was as follows. First, the primary sampling units (i.e., district or county) were selected according to the per capita disposable income (PCDI) of Chinese residents in 2016, i.e., the average PCDI from the lowest quintile to the highest quintile are 5529, 12899, 20924, 31990 and 59209 RMB/year. The Shangrao city has 12 administrative districts/counties, and all districts/counties fell into either the fourth or fifth (the two lowest) quintiles of the average PCDI among Chinese residents. Specifically, two districts/counties were in the fourth PCDI quintile and ten districts/counties were in the fifth PCDI quintile. After ranking the PCDI of the districts/counties in descending order, we sampled one district/county using simple random sampling in the fourth PCDI quintile and three counties in the fifth PCDI quintile. Overall, four districts/counties (i.e., Xinzhou, Yushan, Wuyuan, and Poyang district/county with PCDI being 14358, 12788, 10750, and 8574 RMB/year, respectively.) were selected as primary sampling units. Second, in each district/county selected, two junior high schools and two senior high schools stratified by rural and urban area were randomly selected, with a total of 16 schools. Finally, one class from each grade of the included schools was randomly selected, and all students were invited to take part in the survey.

Through this sampling method, 2704 students were selected, accounting for 1.5% percent of all the junior-senior high school students in the four sampled counties. A total of 2346 students (86.8% response rate) agreed to participate, and 1935 students (49.8% male) were analyzed after data cleaning.

| **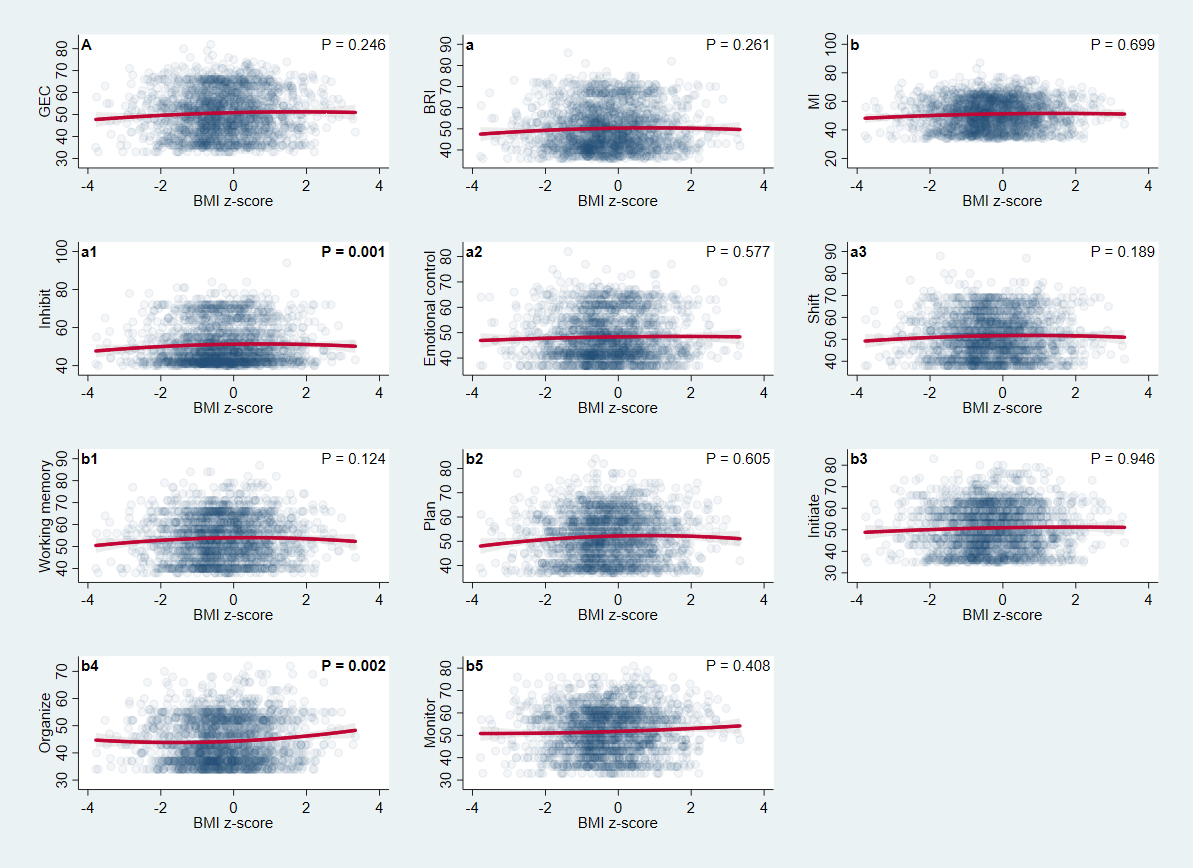** |
| --- |

**Figure S1. Scatter and quadratic fitted plots of BMI z-score and executive function scores in adolescents.**

Note: The P values indicated the statistical significance of the squared BMI z-score on each executive function domain after adjusting for social-demographic factors (i.e. age, sex, parental highest education, and family gross income) and individual lifestyle behaviors (i.e. media exposure, night sleep duration, and physical activity).

Abbreviations: BMI, body mass index; BRI, the Behavioral Regulation Index; GEC, the Global Executive Composite; MI, the Metacognition Index.

| **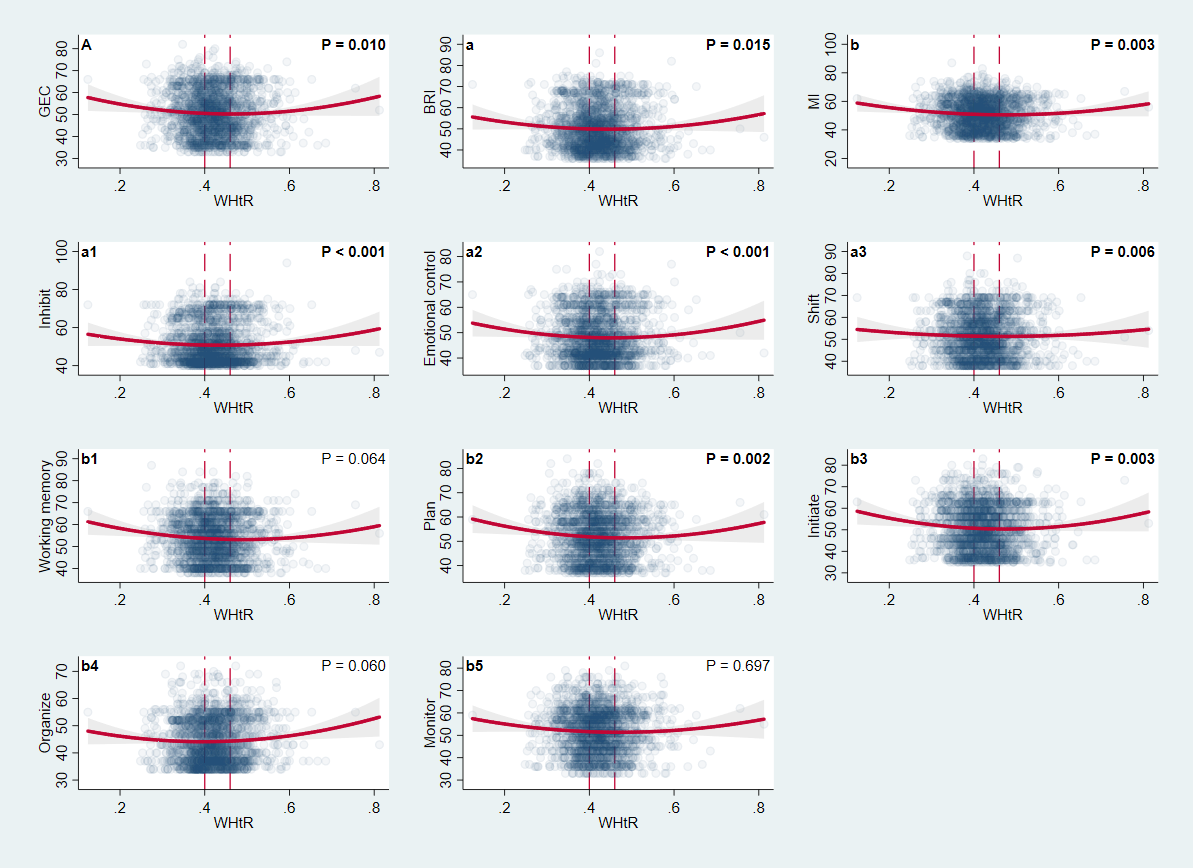** |
| --- |

**Figure S2. Scatter and quadratic fitted plots of WHtR and executive function scores in adolescents.**

Note: The P values indicated the statistical significance of the squared WHtR on each executive function domain after adjusting for social-demographic factors (i.e. age, sex, parental highest education, and family gross income) and individual lifestyle behaviors (i.e. media exposure, night sleep duration, and physical activity); The dash red lines indicated two cutoffs of WHtR, i.e., 0.40 and 0.46.

Abbreviations: BRI, the Behavioral Regulation Index; GEC, the Global Executive Composite; MI, the Metacognition Index; WHtR, waist-to-height ratio.

| **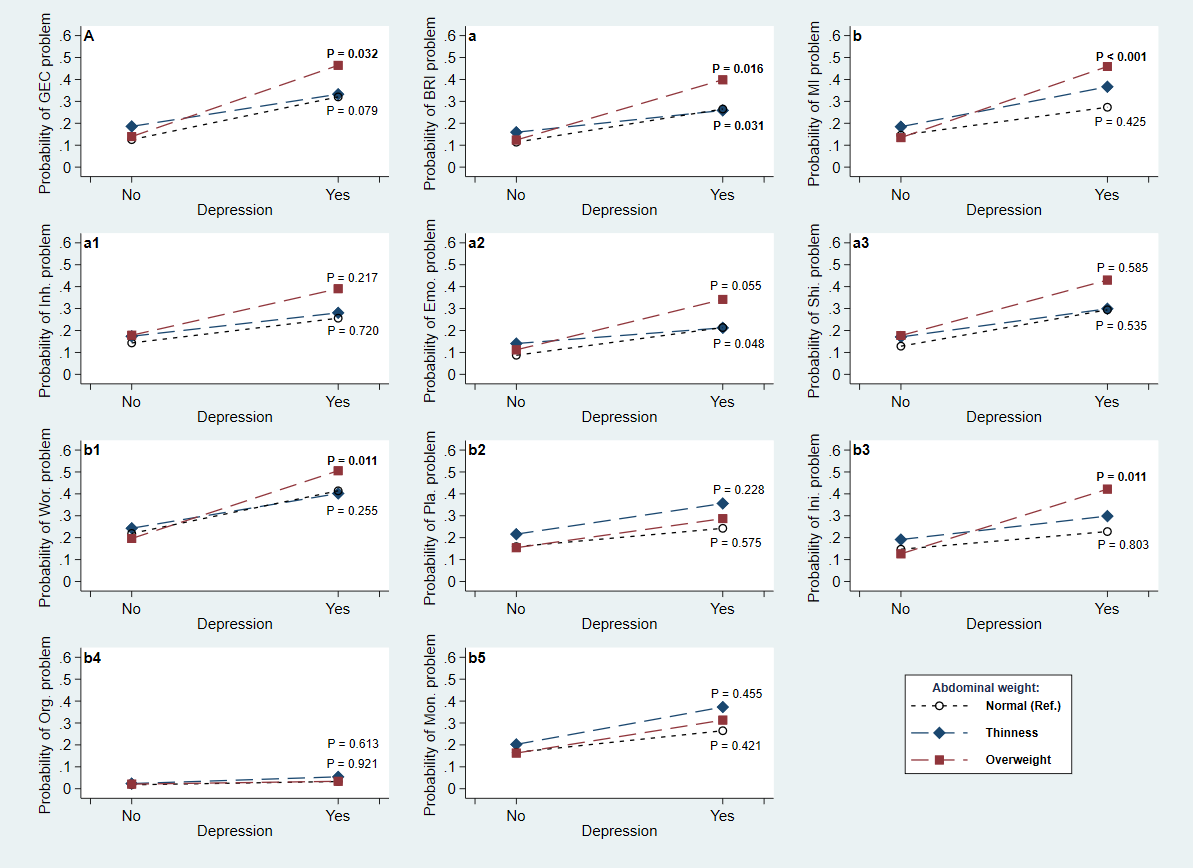** |
| --- |

**Figure S3. Moderating effects of depression condition in the association between abdominal weight spectrum and executive function problems in adolescents.**

Note: All models were adjusted for sociodemographic characteristics (i.e., age, sex, parental education level, and gross family income) and individual behaviors (screen time, night sleep duration, and physical activity), and the P values indicated whether each interaction term of depression and abdominal weight spectrum (i.e., abdominal thinness, and overweight) on executive dysfunction reached statistical significance.

Abbreviations: BRI, the Behavioral Regulation Index; Emo., emotion control domain; GEC, the Global Executive Composite; Ini., initiate domain; Inh., inhibit domain; MI, the Metacognition Index; Mon., monitor domain; Org., organize domain; Pla., plan domain; Shi., shift domain; WHtR, waist-to-height ratio; Wor., working memory domain.

| **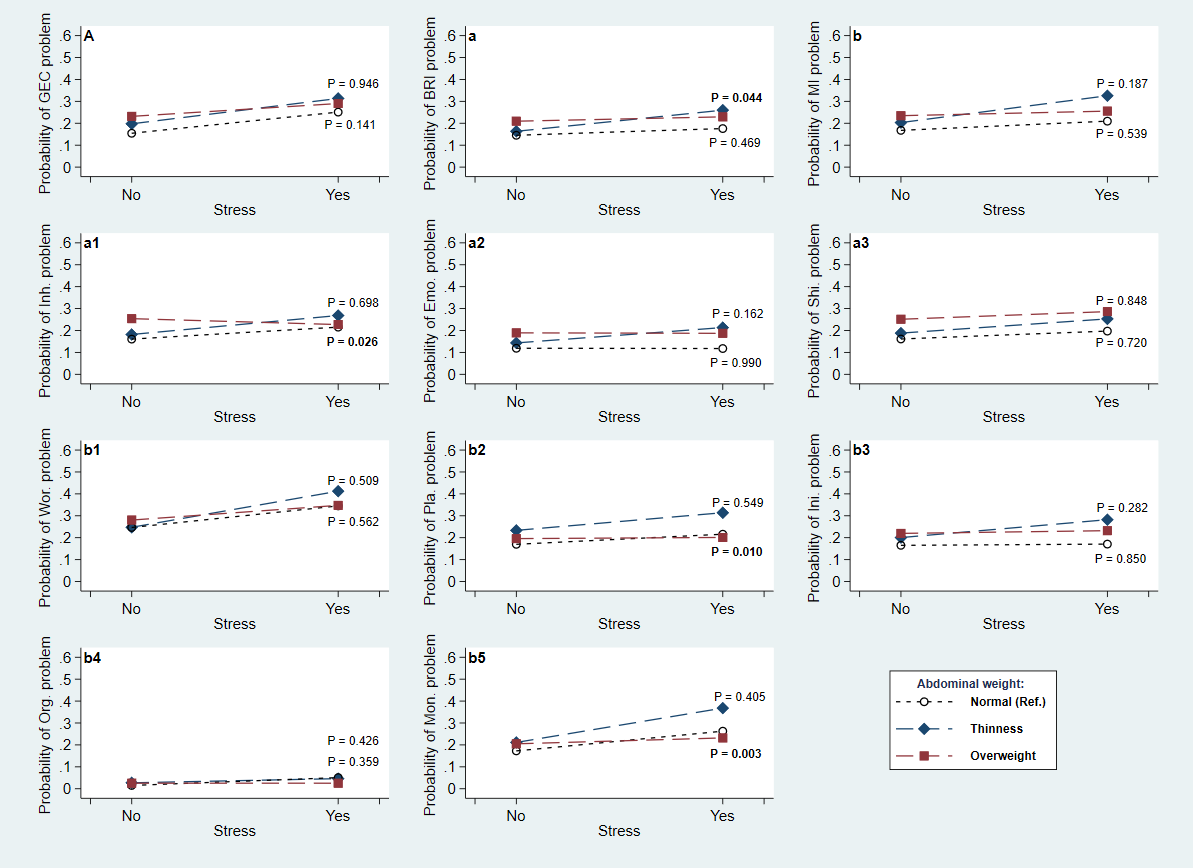** |
| --- |

**Figure S4. Moderating effects of stress condition in the association between abdominal weight spectrum and executive function problems in adolescents.**

Note: All models were adjusted for sociodemographic characteristics (i.e., age, sex, parental education level, and gross family income) and individual behaviors (screen time, night sleep duration, and physical activity), and the P values indicated whether each interaction term of stress and abdominal weight spectrum (i.e., abdominal thinness, and overweight) on executive dysfunction reached statistical significance.

Abbreviations: BRI, the Behavioral Regulation Index; Emo., emotion control domain; GEC, the Global Executive Composite; Ini., initiate domain; Inh., inhibit domain; MI, the Metacognition Index; Mon., monitor domain; Org., organize domain; Pla., plan domain; Shi., shift domain; WHtR, waist-to-height ratio; Wor., working memory domain.

**Table S1.** Participant characteristics between analyzed and excluded sample.

|  | All  (n = 2346) | Analyzed  (n = 1935) | Excluded  (n = 411) | t/χ | P value |
| --- | --- | --- | --- | --- | --- |
| **Age, y** | 15.33 ± 1.77 | 15.32 ± 1.79 | 15.38 ± 1.70 | -0.65 | 0.513 |
| **Sex** |  |  |  | 0.88 | 0.349 |
| Boys | 1178 (50.2) | 963 (49.8) | 215 (52.3) |  |  |
| Girls | 1168 (49.8) | 972 (50.2) | 196 (47.7) |  |  |
| **Household factors** |  |  |  |  |  |
| Parental highest education |  |  |  | 3.49 | 0.062 |
| Lower than high school | 1675 (74.0) | 1374 (73.2) | 301 (77.8) |  |  |
| High school or higher | 589 (26.0) | 503 (26.8) | 86 (22.2) |  |  |
| Family income (RMB) |  |  |  | 0.18 | 0.670 |
| < 50000 | 1125 (58.5) | 939 (58.7) | 186 (57.4) |  |  |
| ≥ 50000 | 799 (41.5) | 661 (41.3) | 138 (42.6) |  |  |
| **Individual behaviors** |  |  |  |  |  |
| Screen exposure |  |  |  |  |  |
| Sitting&Watching, ≥ 2h/day | 1063 (46.4) | 868 (44.9) | 195 (54.6) | 11.50 | **0.001** |
| Playing games, ≥ 2h/day | 950 (41.5) | 769 (39.8) | 181 (50.7) | 14.86 | **< 0.001** |
| Night sleep duration, short | 1148 (50.8) | 978 (51.1) | 170 (49.1) | 0.46 | 0.495 |
| Physical activity |  |  |  | 5.22 | 0.074 |
| Low | 680 (29.7) | 557 (28.8) | 123 (34.5) |  |  |
| Moderate | 824 (36.0) | 710 (36.7) | 114 (31.9) |  |  |
| High | 786 (34.3) | 666 (34.5) | 120 (33.6) |  |  |

The bold words represent P values less that 0.05.

**Table S2.** Association of overall and abdominal weight spectrum, as well as negative emotions with executive function problems in adolescents^1^.

|  | The Global Executive Composite (GEC) | | | | | | | | |
| --- | --- | --- | --- | --- | --- | --- | --- | --- | --- |
|  | The Behavioral Regulation Index (BRI) | | |  | The Metacognition Index (MI) | | | | |
|  | Inhibit | Emotion control | Shift |  | Working memory | Plan | Initiate | Organize | Monitor |
| **Model a-e** |  |  |  |  |  |  |  |  |  |
| **Overall weight status** |  |  |  |  |  |  |  |  |  |
| Normal | Ref. | Ref. | Ref. |  | Ref. | Ref. | Ref. | Ref. | Ref. |
| Overweight | 0.75 (0.52, 1.09) | 0.93 (0.64, 1.36) | 0.92 (0.57, 1.49) |  | 0.91 (0.65, 1.28) | 1.15 (0.49, 2.69) | 1.1 (0.68, 1.79) | 2.49 (0.92, 6.75) | 1.11 (0.86, 1.43) |
| **Abdominal weight spectrum** |  |  |  |  |  |  |  |  |  |
| Normal | Ref. | Ref. | Ref. |  | Ref. | Ref. | Ref. | Ref. | Ref. |
| Thinness | 1.14 (0.69, 1.86) | 1.27 (0.54, 2.97) | 1.26 (0.68, 2.35) |  | 1.13 (0.75, 1.70) | **1.42 (1.07, 1.89)*** | 1.37 (0.88, 2.14) | 1.39 (0.82, 2.36) | **1.27 (1.08, 1.49)**** |
| Overweight | **1.68 (1.15, 2.45)**** | **1.73 (1.05, 2.86)*** | **1.77 (1.07, 2.92)*** |  | **1.25 (1.11, 1.40)***** | 1.20 (0.96, 1.48) | 1.50 (0.92, 2.44) | 1.09 (0.41, 2.88) | **1.13 (1.00, 1.28)*** |
| **Depression** |  |  |  |  |  |  |  |  |  |
| No | Ref. | Ref. | Ref. |  | Ref. | Ref. | Ref. | Ref. | Ref. |
| Yes | **2.34 (1.11, 4.94)*** | **2.75 (1.02, 7.36)*** | **2.86 (1.43, 5.71)**** |  | **2.76 (1.67, 4.57)***** | **1.96 (1.36, 2.83)***** | **2.38 (1.14, 4.96)*** | **1.97 (1.13, 3.44)*** | **2.13 (1.62, 2.79)***** |
| **Anxiety** |  |  |  |  |  |  |  |  |  |
| No | Ref. | Ref. | Ref. |  | Ref. | Ref. | Ref. | Ref. | Ref. |
| Yes | **2.59 (1.97, 3.42)***** | **2.41 (1.36, 4.27)**** | **2.19 (1.52, 3.18)***** |  | **2.43 (1.70, 3.48)***** | **1.76 (1.30, 2.38)***** | **2.21 (1.22, 4.00)**** | 1.79 (0.88, 3.65) | **2.11 (1.52, 2.93)***** |
| **Stress** |  |  |  |  |  |  |  |  |  |
| No | Ref. | Ref. | Ref. |  | Ref. | Ref. | Ref. | Ref. | Ref. |
| Yes | 1.36 (0.82, 2.24) | 1.23 (0.72, 2.11) | 1.35 (0.80, 2.28) |  | **1.75 (1.06, 2.88)*** | 1.34 (0.82, 2.21) | 1.25 (0.94, 1.68) | **2.01 (1.63, 2.49)***** | **1.73 (1.15, 2.6)**** |

^1^ Adjusted for social-demographic factors (i.e. age, sex, parental highest education, and family gross income) and individual lifestyle behaviors (i.e. media exposure, night sleep duration, and physical activity).

The bold words represent the P values less than 0.05.

* *P* < 0.05, ** *P* < 0.01, *** *P* < 0.001.

**Table S3.** Moderating effects of negative emotions in the association of overall and abdominal weight spectrum with executive function problems in adolescents^1^.

|  | GEC problem | | BRI problem | | MI problem | |
| --- | --- | --- | --- | --- | --- | --- |
|  | OR (95% CI) | P value | OR (95% CI) | P value | OR (95% CI) | P value |
| **Model a** |  |  |  |  |  |  |
| Overall overweight | 1.07 (0.77, 1.49) | 0.680 | 0.93 (0.54, 1.61) | 0.804 | 1.04 (0.54, 2.00) | 0.914 |
| Depression | **3.48 (1.47, 8.23)** | **0.005** | **3.00 (1.17, 7.69)** | **0.022** | **2.96 (1.46, 6.00)** | **0.003** |
| Overall overweight×Depression | 0.78 (0.35, 1.74) | 0.545 | 0.87 (0.39, 1.91) | 0.728 | 1.20 (0.52, 2.74) | 0.673 |
| **Model b** |  |  |  |  |  |  |
| Overall overweight | 1.16 (0.56, 2.40) | 0.691 | 0.81 (0.46, 1.43) | 0.469 | 1.40 (0.58, 3.37) | 0.459 |
| Anxiety | **2.86 (1.87, 4.36)** | **< 0.001** | **3.02 (2.06, 4.43)** | **< 0.001** | **2.52 (1.56, 4.09)** | **< 0.001** |
| Overall overweight×Anxiety | 0.78 (0.24, 2.51) | 0.672 | 1.11 (0.30, 4.06) | 0.878 | 0.73 (0.28, 1.89) | 0.521 |
| **Model c** |  |  |  |  |  |  |
| Overall overweight | 1.08 (0.83, 1.42) | 0.558 | 1.02 (0.68, 1.52) | 0.924 | 1.22 (0.69, 2.17) | 0.492 |
| Stress | **1.82 (1.12, 2.94)** | **0.015** | **1.51 (1.04, 2.20)** | **0.032** | 1.52 (1.00, 2.30) | 0.051 |
| Overall overweight×Stress | 0.74 (0.38, 1.43) | 0.367 | 0.65 (0.21, 2.03) | 0.457 | 0.81 (0.53, 1.24) | 0.335 |
| **Model d** |  |  |  |  |  |  |
| Abdominal thinness | **1.56 (1.08, 2.25)** | **0.019** | 1.41 (0.68, 2.93) | 0.356 | 1.29 (0.95, 1.73) | 0.100 |
| Abdominal overweight | 1.15 (0.85, 1.55) | 0.368 | 1.13 (0.81, 1.56) | 0.468 | 0.96 (0.73, 1.25) | 0.756 |
| Depression | **3.39 (1.38, 8.32)** | **0.008** | **2.92 (1.16, 7.30)** | **0.022** | **2.23 (1.16, 4.31)** | **0.017** |
| Abdominal thinness×Depression | 0.66 (0.42, 1.05) | 0.079 | **0.65 (0.44, 0.96)** | **0.031** | 1.16 (0.80, 1.68) | 0.425 |
| Abdominal overweight×Depression | **1.67 (1.04, 2.66)** | **0.032** | **1.72 (1.11, 2.69)** | **0.016** | **2.52 (1.65, 3.84)** | **< 0.001** |
| **Model e** |  |  |  |  |  |  |
| Abdominal thinness | **1.64 (1.07, 2.52)** | **0.023** | 1.77 (0.88, 3.56) | 0.109 | **1.60 (1.36, 1.87)** | **< 0.001** |
| Abdominal overweight | 1.23 (0.84, 1.80) | 0.288 | 1.19 (1.00, 1.43) | 0.050 | 1.17 (0.57, 2.41) | 0.672 |
| Anxiety | **2.94 (1.88, 4.62)** | **< 0.001** | **3.54 (2.37, 5.29)** | **< 0.001** | **2.51 (1.40, 4.52)** | **0.002** |
| Abdominal thinness×Anxiety | 0.74 (0.43, 1.27) | 0.274 | 0.58 (0.25, 1.33) | 0.196 | 0.78 (0.49, 1.24) | 0.287 |
| Abdominal overweight×Anxiety | 1.29 (0.59, 2.83) | 0.520 | 1.31 (0.60, 2.86) | 0.493 | 1.36 (0.54, 3.41) | 0.511 |
| **Model f** |  |  |  |  |  |  |
| Abdominal thinness | 1.30 (0.86, 1.97) | 0.210 | 1.08 (0.51, 2.30) | 0.835 | 1.22 (0.87, 1.69) | 0.246 |
| Abdominal overweight | **1.69 (1.07, 2.67)** | **0.024** | 1.62 (0.93, 2.82) | 0.090 | **1.60 (1.19, 2.15)** | **0.002** |
| Stress | **1.87 (1.01, 3.46)** | **0.046** | 1.27 (0.81, 1.98) | 0.301 | 1.32 (0.83, 2.11) | 0.241 |
| Abdominal thinness×Stress | 1.02 (0.64, 1.60) | 0.946 | **1.46 (1.01, 2.11)** | **0.044** | 1.45 (0.83, 2.52) | 0.187 |
| Abdominal overweight×Stress | 0.73 (0.49, 1.11) | 0.141 | 0.89 (0.65, 1.21) | 0.469 | 0.85 (0.50, 1.43) | 0.539 |

Abbreviations: BRI, the Behavioral Regulation Index; GEC, the Global Executive Composite; MI, the Metacognition Index.

^1^ All models (a to f) were adjusted for social-demographic factors (i.e. age, sex, parental highest education, and family gross income) and individual lifestyle behaviors (i.e. screen exposure, night sleep duration, and physical activity).

The bold words represent the P values less than 0.05.

**Table S4.** Moderating effects of depression in the association between abdominal weight spectrum and executive function problems in adolescents^1^.

|  | The Global Executive Composite (GEC) | | | | | | | | |
| --- | --- | --- | --- | --- | --- | --- | --- | --- | --- |
|  | The Behavioral Regulation Index (BRI) | | |  | The Metacognition Index (MI) | | | | |
|  | Inhibit | Emotion control | Shift |  | Working memory | Plan | Initiate | Organize | Monitor |
| Abdominal thinness | 1.19 (0.74, 1.93) | 1.58 (0.69, 3.61) | 1.43 (0.73, 2.80) |  | 1.21 (0.84, 1.73) | **1.35 (1.02, 1.79)*** | 1.36 (0.83, 2.22) | 1.27 (0.95, 1.70) | 1.18 (0.87, 1.60) |
| Abdominal overweight | 1.35 (0.97, 1.89) | 1.31 (0.90, 1.91) | 1.48 (0.91, 2.43) |  | 0.93 (0.75, 1.16) | 1.02 (0.57, 1.83) | 0.87 (0.47, 1.63) | 1.08 (0.68, 1.72) | 0.96 (0.80, 1.15) |
| Depression | 2.12 (0.91, 4.97) | **2.93 (1.20, 7.16)*** | **2.91 (1.42, 5.96)**** |  | **2.57 (1.46, 4.52)**** | **1.72 (1.22, 2.44)**** | 1.72 (0.99, 3.00) | 1.85 (0.60, 5.76) | **1.82 (1.27, 2.62)**** |
| Abdominal thinness×Depression | 0.90 (0.51, 1.59) | **0.57 (0.33, 0.99)*** | 0.73 (0.27, 1.97) |  | 0.83 (0.60, 1.15) | 1.19 (0.89, 1.59) | 1.05 (0.69, 1.60) | 1.29 (0.48, 3.45) | 1.30 (0.65, 2.59) |
| Abdominal overweight×Depression | 1.47 (0.80, 2.71) | 1.49 (0.99, 2.24) | 1.26 (0.55, 2.91) |  | **1.68 (1.13, 2.52)*** | 1.32 (0.50, 3.45) | **3.01 (1.29, 7.03)*** | 0.88 (0.07, 10.49) | 1.30 (0.69, 2.45) |

^1^ Adjusted for social-demographic factors (i.e. age, sex, parental highest education, and family gross income) and individual lifestyle behaviors (i.e. screen exposure, night sleep duration, and physical activity).

The bold words represent the P values less than 0.05.

* *P* < 0.05, ** *P* < 0.01, *** *P* < 0.001.

**Table S5.** Simple effects of abdominal weight spectrum on executive function problems stratified by depression condition in adolescents^1^.

|  | The Global Executive Composite (GEC) | | | | | | | | |
| --- | --- | --- | --- | --- | --- | --- | --- | --- | --- |
|  | The Behavioral Regulation Index (BRI) | | |  | The Metacognition Index (MI) | | | | |
|  | Inhibit | Emotion control | Shift |  | Working memory | Plan | Initiate | Organize | Monitor |
| **No depression** |  |  |  |  |  |  |  |  |  |
| Abdominal weight spectrum |  |  |  |  |  |  |  |  |  |
| Normal | Ref. | Ref. | Ref. |  | Ref. | Ref. | Ref. | Ref. | Ref. |
| Thinness | 1.16 (0.72, 1.88) | 1.56 (0.71, 3.41) | 1.42 (0.76, 2.67) |  | 1.21 (0.85, 1.71) | **1.38 (1.03, 1.83)*** | 1.36 (0.85, 2.19) | 1.36 (1.00, 1.86) | 1.18 (0.88, 1.59) |
| Overweight | 1.33 (0.88, 2.00) | 1.28 (0.84, 1.97) | 1.42 (0.81, 2.51) |  | 0.92 (0.75, 1.13) | 1.05 (0.60, 1.82) | 0.85 (0.46, 1.60) | 1.06 (0.60, 1.87) | 0.97 (0.82, 1.14) |
| **Depression** |  |  |  |  |  |  |  |  |  |
| Abdominal weight spectrum |  |  |  |  |  |  |  |  |  |
| Normal | Ref. | Ref. | Ref. |  | Ref. | Ref. | Ref. | Ref. | Ref. |
| Thinness | 1.09 (0.56, 2.13) | 0.90 (0.33, 2.44) | 1.04 (0.38, 2.87) |  | 0.99 (0.48, 2.05) | 1.57 (0.86, 2.88) | 1.46 (0.92, 2.31) | 1.43 (0.50, 4.11) | 1.45 (0.87, 2.41) |
| Overweight | **2.13 (1.29, 3.51)**** | **2.15 (1.42, 3.25)***** | **2.08 (1.04, 4.15)*** |  | **1.69 (1.21, 2.35)**** | 1.31 (0.79, 2.18) | **2.92 (1.64, 5.19)***** | 1.05 (0.09, 12.05) | 1.25 (0.67, 2.32) |

^1^ Adjusted for social-demographic factors (i.e. age, sex, parental highest education, and family gross income) and individual lifestyle behaviors (i.e. screen exposure, night sleep duration, and physical activity).

The bold words represent the P values less than 0.05.

* *P* < 0.05, ** *P* < 0.01, *** *P* < 0.001.

**Table S6.** Association of overall and abdominal weight spectrum, as well as negative emotions with executive function problems in adolescents.

|  | GEC problem | | BRI problem | | MI problem | |
| --- | --- | --- | --- | --- | --- | --- |
|  | OR (95% CI) | P value | OR (95% CI) | P value | OR (95% CI) | P value |
| **Model a-e**^1^ |  |  |  |  |  |  |
| **Overall weight status** |  |  |  |  |  |  |
| Normal | Ref. |  | Ref. |  | Ref. |  |
| Overweight | 1.02 (0.65, 1.58) | 0.939 | 0.93 (0.53, 1.62) | 0.795 | 1.18 (0.64, 2.17) | 0.605 |
| **Abdominal weight spectrum** |  |  |  |  |  |  |
| Normal | Ref. |  | Ref. |  | Ref. |  |
| Thinness | 1.30 (0.86, 1.97) | 0.210 | 1.18 (0.56, 2.50) | 0.670 | **1.32 (1.01, 1.73)** | **0.042** |
| Overweight | **1.59 (1.08, 2.35)** | **0.018** | 1.59 (0.94, 2.70) | 0.085 | **1.56 (1.22, 1.99)** | **< 0.001** |
| **Depression** |  |  |  |  |  |  |
| No | Ref. |  | Ref. |  | Ref. |  |
| Yes | **3.35 (1.35, 8.34)** | **0.009** | **2.93 (1.08, 7.97)** | **0.035** | **3.01 (1.45, 6.26)** | **0.003** |
| **Anxiety** |  |  |  |  |  |  |
| No | Ref. |  | Ref. |  | Ref. |  |
| Yes | **2.78 (1.66, 4.65)** | **< 0.001** | **3.03 (1.86, 4.94)** | **< 0.001** | **2.44 (1.37, 4.34)** | **0.003** |
| **Stress** |  |  |  |  |  |  |
| No | Ref. |  | Ref. |  | Ref. |  |
| Yes | **1.76 (1.07, 2.87)** | **0.025** | **1.44 (1.05, 1.98)** | **0.024** | 1.49 (0.96, 2.30) | 0.074 |

Abbreviations: BRI, the Behavioral Regulation Index; GEC, the Global Executive Composite; MI, the Metacognition Index.

^1^ All models were adjusted for social-demographic factors (i.e. age, sex, parental highest education, and family gross income) and individual lifestyle behaviors (i.e. screen exposure, night sleep duration, and physical activity).

The bold words represent the P values less than 0.05.

**Table S7.** Associations of negative emotions and abdominal weight spectrum in adolescents^1^.

|  | Abdominal thinness vs normal | | Abdominal overweight vs normal | |
| --- | --- | --- | --- | --- |
|  | RRR (95% CI) | P value | RRR (95% CI) | P value |
| Depression | 0.95 (0.75, 1.19) | 0.644 | **1.43 (1.04, 1.97)** | **0.026** |
| Anxiety | 0.90 (0.76, 1.08) | 0.256 | 1.19 (0.99, 1.44) | 0.057 |
| Stress | 1.17 (0.74, 1.84) | 0.503 | 0.96 (0.69, 1.34) | 0.827 |

Abbreviations: RRR, relative risk ratio.

^1^ The model was adjusted for social-demographic factors (i.e. age, sex, parental highest education, and family gross income) and individual lifestyle behaviors (i.e. screen exposure, night sleep duration, and physical activity).

The bold words represent the P values less than 0.05.

**Table S8.** Association of overall and abdominal weight spectrum, as well as negative emotions with executive function problems in adolescents^1^.

|  | The Global Executive Composite (GEC) | | | | | | | | |
| --- | --- | --- | --- | --- | --- | --- | --- | --- | --- |
|  | The Behavioral Regulation Index (BRI) | | |  | The Metacognition Index (MI) | | | | |
|  | Inhibit | Emotion control | Shift |  | Working memory | Plan | Initiate | Organize | Monitor |
| **Model a-e** |  |  |  |  |  |  |  |  |  |
| **Overall weight status** |  |  |  |  |  |  |  |  |  |
| Normal | Ref. | Ref. | Ref. |  | Ref. | Ref. | Ref. | Ref. | Ref. |
| Overweight | 0.76 (0.52, 1.09) | 0.94 (0.64, 1.37) | 0.93 (0.57, 1.50) |  | 0.91 (0.65, 1.28) | 1.15 (0.49, 2.69) | 1.11 (0.68, 1.79) | 2.49 (0.92, 6.75) | 1.11 (0.86, 1.43) |
| **Abdominal weight spectrum** |  |  |  |  |  |  |  |  |  |
| Normal | Ref. | Ref. | Ref. |  | Ref. | Ref. | Ref. | Ref. | Ref. |
| Thinness | 1.13 (0.68, 1.87) | 1.25 (0.52, 2.99) | 1.25 (0.66, 2.38) |  | 1.12 (0.74, 1.70) | **1.42 (1.07, 1.89)*** | 1.36 (0.86, 2.16) | 1.39 (0.82, 2.37) | **1.27 (1.08, 1.49)**** |
| Overweight | **1.68 (1.16, 2.44)**** | **1.74 (1.06, 2.86)*** | **1.77 (1.08, 2.91)*** |  | **1.25 (1.11, 1.41)***** | 1.18 (0.93, 1.49) | 1.50 (0.93, 2.44) | 1.09 (0.41, 2.89) | **1.14 (1.01, 1.28)*** |
| **Depression** |  |  |  |  |  |  |  |  |  |
| No | Ref. | Ref. | Ref. |  | Ref. | Ref. | Ref. | Ref. | Ref. |
| Yes | **2.33 (1.11, 4.86)*** | **2.72 (1.03, 7.22)*** | **2.84 (1.43, 5.64)**** |  | **2.74 (1.66, 4.53)***** | **1.97 (1.37, 2.85)***** | **2.36 (1.14, 4.87)*** | **1.97 (1.13, 3.44)*** | **2.13 (1.62, 2.81)***** |
| **Anxiety** |  |  |  |  |  |  |  |  |  |
| No | Ref. | Ref. | Ref. |  | Ref. | Ref. | Ref. | Ref. | Ref. |
| Yes | **2.59 (1.98, 3.38)***** | **2.4 (1.37, 4.21)**** | **2.19 (1.52, 3.14)***** |  | **2.43 (1.71, 3.45)***** | **1.75 (1.29, 2.39)***** | **2.21 (1.23, 3.96)**** | 1.79 (0.88, 3.66) | **2.12 (1.52, 2.94)***** |
| **Stress** |  |  |  |  |  |  |  |  |  |
| No | Ref. | Ref. | Ref. |  | Ref. | Ref. | Ref. | Ref. | Ref. |
| Yes | 1.36 (0.83, 2.24) | 1.24 (0.72, 2.11) | 1.36 (0.81, 2.28) |  | **1.75 (1.06, 2.89)*** | 1.35 (0.82, 2.21) | 1.26 (0.94, 1.68) | **2.01 (1.63, 2.48)***** | **1.73 (1.15, 2.6)**** |

^1^ Adjusted for social-demographic factors (i.e. age, sex, parental highest education, and family gross income) and individual lifestyle behaviors (i.e. media exposure, night sleep duration, and physical activity).

The bold words represent the P values less than 0.05.

* *P* < 0.05, ** *P* < 0.01, *** *P* < 0.001.

**Table S9.** Moderating effects of negative emotions in the association of overall and abdominal weight spectrum with executive function problems in adolescents^1^.

|  | GEC problem | | BRI problem | | MI problem | |
| --- | --- | --- | --- | --- | --- | --- |
|  | OR (95% CI) | P value | OR (95% CI) | P value | OR (95% CI) | P value |
| **Model a** |  |  |  |  |  |  |
| Overall overweight | 1.07 (0.77, 1.49) | 0.683 | 0.93 (0.54, 1.61) | 0.803 | 1.04 (0.54, 2.00) | 0.916 |
| Depression | **3.45 (1.47, 8.12)** | **0.005** | **2.98 (1.17, 7.56)** | **0.022** | **2.94 (1.47, 5.90)** | **0.002** |
| Overall overweight×Depression | 0.79 (0.36, 1.74) | 0.555 | 0.88 (0.40, 1.93) | 0.744 | 1.21 (0.53, 2.77) | 0.657 |
| **Model b** |  |  |  |  |  |  |
| Overall overweight | 1.16 (0.56, 2.41) | 0.690 | 0.81 (0.46, 1.44) | 0.474 | 1.40 (0.58, 3.37) | 0.457 |
| Anxiety | **2.85 (1.89, 4.31)** | **< 0.001** | **3.01 (2.08, 4.37)** | **< 0.001** | **2.52 (1.57, 4.04)** | **< 0.001** |
| Overall overweight×Anxiety | 0.78 (0.24, 2.53) | 0.677 | 1.11 (0.30, 4.10) | 0.875 | 0.74 (0.28, 1.91) | 0.529 |
| **Model c** |  |  |  |  |  |  |
| Overall overweight | 1.09 (0.83, 1.42) | 0.541 | 1.02 (0.68, 1.54) | 0.907 | 1.23 (0.69, 2.17) | 0.481 |
| Stress | **1.82 (1.13, 2.94)** | **0.014** | **1.51 (1.04, 2.20)** | **0.029** | 1.52 (1.00, 2.31) | 0.049 |
| Overall overweight×Stress | 0.73 (0.38, 1.42) | 0.360 | 0.65 (0.21, 2.02) | 0.453 | 0.81 (0.53, 1.23) | 0.324 |
| **Model d** |  |  |  |  |  |  |
| Abdominal thinness | **1.56 (1.07, 2.25)** | **0.020** | 1.41 (0.68, 2.93) | 0.358 | 1.29 (0.95, 1.74) | 0.102 |
| Abdominal overweight | 1.15 (0.86, 1.55) | 0.349 | 1.13 (0.82, 1.56) | 0.452 | 0.96 (0.73, 1.27) | 0.791 |
| Depression | **3.39 (1.38, 8.32)** | **0.008** | **2.92 (1.17, 7.30)** | **0.022** | **2.23 (1.16, 4.31)** | **0.017** |
| Abdominal thinness×Depression | 0.65 (0.40, 1.04) | 0.072 | **0.63 (0.42, 0.94)** | **0.024** | 1.14 (0.78, 1.65) | 0.495 |
| Abdominal overweight×Depression | **1.66 (1.04, 2.65)** | **0.035** | **1.72 (1.10, 2.68)** | **0.017** | **2.50 (1.63, 3.85)** | **< 0.001** |
| **Model e** |  |  |  |  |  |  |
| Abdominal thinness | **1.64 (1.07, 2.52)** | **0.023** | 1.77 (0.88, 3.57) | 0.111 | **1.60 (1.36, 1.87)** | **< 0.001** |
| Abdominal overweight | 1.23 (0.84, 1.80) | 0.294 | 1.19 (1.00, 1.43) | 0.054 | 1.17 (0.56, 2.42) | 0.674 |
| Anxiety | **2.95 (1.88, 4.61)** | **< 0.001** | **3.54 (2.38, 5.28)** | **< 0.001** | **2.51 (1.40, 4.52)** | **0.002** |
| Abdominal thinness×Anxiety | 0.73 (0.42, 1.26) | 0.258 | 0.57 (0.25, 1.30) | 0.183 | 0.77 (0.47, 1.25) | 0.285 |
| Abdominal overweight×Anxiety | 1.30 (0.60, 2.85) | 0.507 | 1.32 (0.61, 2.87) | 0.484 | 1.37 (0.55, 3.42) | 0.496 |
| **Model f** |  |  |  |  |  |  |
| Abdominal thinness | 1.29 (0.83, 2.00) | 0.255 | 1.07 (0.49, 2.33) | 0.866 | 1.20 (0.84, 1.71) | 0.307 |
| Abdominal overweight | **1.70 (1.08, 2.66)** | **0.021** | 1.62 (0.94, 2.81) | 0.084 | **1.61 (1.19, 2.17)** | **0.002** |
| Stress | **1.87 (1.01, 3.46)** | **0.046** | 1.27 (0.81, 1.98) | 0.301 | 1.32 (0.83, 2.11) | 0.241 |
| Abdominal thinness×Stress | 1.03 (0.65, 1.61) | 0.912 | **1.48 (1.01, 2.15)** | **0.043** | 1.47 (0.84, 2.56) | 0.180 |
| Abdominal overweight×Stress | 0.73 (0.49, 1.10) | 0.129 | 0.89 (0.66, 1.20) | 0.446 | 0.85 (0.50, 1.43) | 0.533 |

Abbreviations: BRI, the Behavioral Regulation Index; GEC, the Global Executive Composite; MI, the Metacognition Index.

^1^ All models (a to f) were adjusted for social-demographic factors (i.e. age, sex, parental highest education, and family gross income) and individual lifestyle behaviors (i.e. screen exposure, night sleep duration, and physical activity).

The bold words represent the P values less than 0.05.

**Table S10.** Moderating effects of depression in the association between abdominal weight spectrum and executive function problems in adolescents^1^.

|  | The Global Executive Composite (GEC) | | | | | | | | |
| --- | --- | --- | --- | --- | --- | --- | --- | --- | --- |
|  | The Behavioral Regulation Index (BRI) | | |  | The Metacognition Index (MI) | | | | |
|  | Inhibit | Emotion control | Shift |  | Working memory | Plan | Initiate | Organize | Monitor |
| Abdominal thinness | 1.19 (0.74, 1.93) | 1.57 (0.69, 3.61) | 1.43 (0.73, 2.80) |  | 1.21 (0.84, 1.74) | **1.35 (1.02, 1.79)*** | 1.36 (0.83, 2.22) | 1.27 (0.95, 1.70) | 1.18 (0.87, 1.60) |
| Abdominal overweight | 1.36 (0.98, 1.89) | 1.31 (0.90, 1.92) | 1.49 (0.91, 2.43) |  | 0.94 (0.75, 1.17) | 0.99 (0.54, 1.81) | 0.88 (0.47, 1.65) | 1.08 (0.68, 1.74) | 0.97 (0.81, 1.16) |
| Depression | 2.12 (0.91, 4.97) | **2.93 (1.20, 7.18)*** | **2.92 (1.43, 5.96)**** |  | **2.57 (1.46, 4.52)**** | **1.72 (1.21, 2.44)**** | 1.73 (0.99, 3.00) | 1.85 (0.60, 5.76) | **1.82 (1.27, 2.62)**** |
| Abdominal thinness×Depression | 0.88 (0.49, 1.58) | **0.55 (0.31, 1.00)*** | 0.71 (0.26, 1.97) |  | 0.81 (0.58, 1.15) | 1.21 (0.91, 1.61) | 1.03 (0.68, 1.57) | 1.30 (0.48, 3.51) | 1.32 (0.65, 2.66) |
| Abdominal overweight×Depression | 1.46 (0.79, 2.70) | 1.48 (0.99, 2.23) | 1.26 (0.54, 2.90) |  | **1.67 (1.12, 2.51)*** | 1.35 (0.51, 3.55) | **2.99 (1.28, 7.02)*** | 0.88 (0.07, 10.52) | 1.30 (0.69, 2.43) |

^1^ Adjusted for social-demographic factors (i.e. age, sex, parental highest education, and family gross income) and individual lifestyle behaviors (i.e. screen exposure, night sleep duration, and physical activity).

The bold words represent the P values less than 0.05.

* *P* < 0.05, ** *P* < 0.01, *** *P* < 0.001.

**Table S11.** Simple effects of abdominal weight spectrum on executive function problems stratified by depression condition in adolescents^1^.

|  | GEC problem | | BRI problem | | MI problem | |
| --- | --- | --- | --- | --- | --- | --- |
|  | OR (95% CI) | P value | OR (95% CI) | P value | OR (95% CI) | P value |
| **No depression** |  |  |  |  |  |  |
| Abdominal weight spectrum |  |  |  |  |  |  |
| Normal | Ref. |  | Ref. |  | Ref. |  |
| Thinness | **1.51 (1.10, 2.08)** | **0.010** | 1.37 (0.67, 2.80) | 0.386 | **1.30 (1.01, 1.66)** | **0.042** |
| Overweight | 1.14 (0.80, 1.63) | 0.455 | 1.10 (0.73, 1.64) | 0.654 | 0.96 (0.72, 1.28) | 0.787 |
| **Depression** |  |  |  |  |  |  |
| Abdominal weight spectrum |  |  |  |  |  |  |
| Normal | Ref. |  | Ref. |  | Ref. |  |
| Thinness | 0.99 (0.50, 1.96) | 0.973 | 0.92 (0.39, 2.15) | 0.841 | 1.43 (0.86, 2.37) | 0.167 |
| Overweight | **1.91 (1.26, 2.91)** | **0.002** | **2.16 (1.30, 3.60)** | **0.003** | **2.43 (1.82, 3.25)** | **0.000** |

Abbreviations: BRI, the Behavioral Regulation Index; GEC, the Global Executive Composite; MI, the Metacognition Index.

^1^ Adjusted for social-demographic factors (i.e. age, sex, parental highest education, and family gross income) and individual lifestyle behaviors (i.e. screen exposure, night sleep duration, and physical activity).

The bold words represent the P values less than 0.05.

**Table S12.** Simple effects of abdominal weight spectrum on executive function problems stratified by depression condition in adolescents^1^.

|  | The Global Executive Composite (GEC) | | | | | | | | |
| --- | --- | --- | --- | --- | --- | --- | --- | --- | --- |
|  | The Behavioral Regulation Index (BRI) | | |  | The Metacognition Index (MI) | | | | |
|  | Inhibit | Emotion control | Shift |  | Working memory | Plan | Initiate | Organize | Monitor |
| **No depression** |  |  |  |  |  |  |  |  |  |
| Abdominal weight spectrum |  |  |  |  |  |  |  |  |  |
| Normal | Ref. | Ref. | Ref. |  | Ref. | Ref. | Ref. | Ref. | Ref. |
| Thinness | 1.16 (0.72, 1.88) | 1.56 (0.71, 3.41) | 1.42 (0.76, 2.67) |  | 1.21 (0.85, 1.71) | **1.38 (1.03, 1.83)*** | 1.36 (0.85, 2.19) | 1.36 (1.00, 1.86) | 1.18 (0.88, 1.59) |
| Overweight | 1.33 (0.89, 1.99) | 1.29 (0.84, 1.97) | 1.43 (0.81, 2.51) |  | 0.92 (0.74, 1.14) | 1.02 (0.57, 1.80) | 0.86 (0.46, 1.60) | 1.07 (0.60, 1.88) | 0.97 (0.82, 1.15) |
| **Depression** |  |  |  |  |  |  |  |  |  |
| Abdominal weight spectrum |  |  |  |  |  |  |  |  |  |
| Normal | Ref. | Ref. | Ref. |  | Ref. | Ref. | Ref. | Ref. | Ref. |
| Thinness | 1.07 (0.53, 2.14) | 0.87 (0.30, 2.47) | 1.01 (0.35, 2.93) |  | 0.97 (0.46, 2.04) | 1.59 (0.87, 2.88) | 1.42 (0.87, 2.32) | 1.44 (0.50, 4.14) | 1.47 (0.88, 2.44) |
| Overweight | **2.12 (1.29, 3.48)**** | **2.14 (1.42, 3.21)***** | **2.07 (1.04, 4.11)*** |  | **1.68 (1.21, 2.34)**** | 1.31 (0.79, 2.18) | **2.90 (1.65, 5.13)***** | 1.05 (0.09, 12.05) | 1.25 (0.67, 2.32) |

^1^ Adjusted for social-demographic factors (i.e. age, sex, parental highest education, and family gross income) and individual lifestyle behaviors (i.e. screen exposure, night sleep duration, and physical activity).

The bold words represent the P values less than 0.05.

* *P* < 0.05, ** *P* < 0.01, *** *P* < 0.001.

**Table S13.** Association of overall and abdominal weight spectrum, as well as negative emotions with executive function problems in adolescents.

|  | GEC problem | | BRI problem | | MI problem | |
| --- | --- | --- | --- | --- | --- | --- |
|  | OR (95% CI) | P value | OR (95% CI) | P value | OR (95% CI) | P value |
| **Model a-e**^1^ |  |  |  |  |  |  |
| **Overall weight status** |  |  |  |  |  |  |
| Normal | Ref. |  | Ref. |  | Ref. |  |
| Overweight | 0.95 (0.60, 1.50) | 0.815 | 0.95 (0.55, 1.63) | 0.842 | 1.10 (0.59, 2.07) | 0.758 |
| **Abdominal weight spectrum** |  |  |  |  |  |  |
| Normal | Ref. |  | Ref. |  | Ref. |  |
| Thinness | 1.28 (0.79, 2.10) | 0.319 | 1.15 (0.50, 2.61) | 0.741 | 1.27 (0.92, 1.76) | 0.151 |
| Overweight | **1.56 (1.04, 2.34)** | **0.031** | 1.58 (0.96, 2.57) | 0.070 | **1.47 (1.16, 1.85)** | **0.001** |
| **Depression** |  |  |  |  |  |  |
| No | Ref. |  | Ref. |  | Ref. |  |
| Yes | **3.43 (1.30, 9.06)** | **0.013** | **2.94 (1.01, 8.56)** | **0.047** | **3.17 (1.54, 6.53)** | **0.002** |
| **Anxiety** |  |  |  |  |  |  |
| No | Ref. |  | Ref. |  | Ref. |  |
| Yes | **2.68 (1.50, 4.77)** | **0.001** | **2.91 (1.66, 5.10)** | **< 0.001** | **2.42 (1.32, 4.44)** | **0.004** |
| **Stress** |  |  |  |  |  |  |
| No | Ref. |  | Ref. |  | Ref. |  |
| Yes | **1.80 (1.06, 3.07)** | **0.031** | **1.46 (1.06, 2.01)** | **0.022** | **1.58 (1.03, 2.43)** | **0.036** |

Abbreviations: BRI, the Behavioral Regulation Index; GEC, the Global Executive Composite; MI, the Metacognition Index.

^1^ All models were adjusted for social-demographic factors (i.e. age, sex, parental highest education, and family gross income) and individual lifestyle behaviors (i.e. screen exposure, night sleep duration, and physical activity).

The bold words represent the P values less than 0.05.

**Table S14.** Association of overall and abdominal weight spectrum, as well as negative emotions with executive function problems in adolescents^1^.

|  | The Global Executive Composite (GEC) | | | | | | | | |
| --- | --- | --- | --- | --- | --- | --- | --- | --- | --- |
|  | The Behavioral Regulation Index (BRI) | | |  | The Metacognition Index (MI) | | | | |
|  | Inhibit | Emotion control | Shift |  | Working memory | Plan | Initiate | Organize | Monitor |
| **Model a-e** |  |  |  |  |  |  |  |  |  |
| **Overall weight status** |  |  |  |  |  |  |  |  |  |
| Normal | Ref. | Ref. | Ref. |  | Ref. | Ref. | Ref. | Ref. | Ref. |
| Overweight | 0.77 (0.53, 1.11) | 0.91 (0.60, 1.37) | 0.92 (0.62, 1.36) |  | 0.90 (0.60, 1.35) | 1.12 (0.44, 2.87) | 1.05 (0.65, 1.70) | 2.47 (0.93, 6.57) | 1.07 (0.79, 1.47) |
| **Abdominal weight spectrum** |  |  |  |  |  |  |  |  |  |
| Normal | Ref. | Ref. | Ref. |  | Ref. | Ref. | Ref. | Ref. | Ref. |
| Thinness | 1.11 (0.62, 1.97) | 1.20 (0.48, 3.05) | 1.24 (0.59, 2.61) |  | 1.14 (0.73, 1.78) | **1.42 (1.07, 1.88)*** | 1.38 (0.79, 2.42) | 1.31 (0.70, 2.43) | 1.23 (1.00, 1.51) |
| Overweight | **1.68 (1.17, 2.42)**** | **1.65 (1.03, 2.66)*** | **1.76 (1.09, 2.84)*** |  | **1.27 (1.24, 1.31)***** | 1.22 (0.93, 1.58) | 1.48 (0.94, 2.32) | 1.07 (0.39, 2.98) | **1.12 (1.01, 1.25)*** |
| **Depression** |  |  |  |  |  |  |  |  |  |
| No | Ref. | Ref. | Ref. |  | Ref. | Ref. | Ref. | Ref. | Ref. |
| Yes | **2.33 (1.07, 5.04)*** | **2.84 (1.03, 7.88)*** | **2.85 (1.43, 5.68)**** |  | **2.85 (1.69, 4.80)***** | **2.04 (1.32, 3.14)**** | **2.41 (1.17, 4.94)*** | **1.87 (1.06, 3.31)*** | **2.27 (1.79, 2.88)***** |
| **Anxiety** |  |  |  |  |  |  |  |  |  |
| No | Ref. | Ref. | Ref. |  | Ref. | Ref. | Ref. | Ref. | Ref. |
| Yes | **2.47 (1.77, 3.45)***** | **2.5 (1.40, 4.47)**** | **2.08 (1.44, 3.01)***** |  | **2.43 (1.62, 3.65)***** | **1.83 (1.34, 2.49)***** | **2.17 (1.15, 4.08)*** | 1.77 (0.92, 3.43) | **2.12 (1.49, 3.01)***** |
| **Stress** |  |  |  |  |  |  |  |  |  |
| No | Ref. | Ref. | Ref. |  | Ref. | Ref. | Ref. | Ref. | Ref. |
| Yes | 1.35 (0.79, 2.31) | 1.23 (0.73, 2.08) | 1.41 (0.80, 2.46) |  | **1.80 (1.05, 3.10)*** | 1.42 (0.86, 2.34) | 1.30 (0.97, 1.73) | **1.90 (1.60, 2.26)***** | **1.88 (1.26, 2.8)**** |

^1^ Adjusted for social-demographic factors (i.e. age, sex, parental highest education, and family gross income) and individual lifestyle behaviors (i.e. media exposure, night sleep duration, and physical activity).

The bold words represent the P values less than 0.05.

* *P* < 0.05, ** *P* < 0.01, *** *P* < 0.001.

**Table S15.** Moderating effects of negative emotions in the association of overall and abdominal weight spectrum with executive function problems in adolescents^1^.

|  | GEC problem | | BRI problem | | MI problem | |
| --- | --- | --- | --- | --- | --- | --- |
|  | OR (95% CI) | P value | OR (95% CI) | P value | OR (95% CI) | P value |
| **Model a** |  |  |  |  |  |  |
| Overall overweight | 1.01 (0.78, 1.31) | 0.916 | 0.95 (0.56, 1.63) | 0.862 | 1.00 (0.55, 1.82) | 0.997 |
| Depression | **3.56 (1.46, 8.69)** | **0.005** | **3.00 (1.10, 8.17)** | **0.032** | **3.13 (1.60, 6.11)** | **0.001** |
| Overall overweight×Depression | 0.75 (0.30, 1.88) | 0.534 | 0.87 (0.44, 1.71) | 0.686 | 1.11 (0.43, 2.85) | 0.824 |
| **Model b** |  |  |  |  |  |  |
| Overall overweight | 0.99 (0.50, 1.98) | 0.986 | 0.80 (0.48, 1.34) | 0.395 | 1.27 (0.48, 3.40) | 0.630 |
| Anxiety | **2.72 (1.68, 4.42)** | **< 0.001** | **2.87 (1.84, 4.48)** | **< 0.001** | **2.49 (1.51, 4.12)** | **< 0.001** |
| Overall overweight×Anxiety | 0.87 (0.31, 2.44) | 0.792 | 1.16 (0.34, 3.98) | 0.809 | 0.76 (0.28, 2.06) | 0.590 |
| **Model c** |  |  |  |  |  |  |
| Overall overweight | 0.99 (0.74, 1.33) | 0.946 | 1.05 (0.70, 1.57) | 0.806 | 1.15 (0.64, 2.07) | 0.635 |
| Stress | **1.86 (1.11, 3.10)** | **0.018** | **1.55 (1.06, 2.26)** | **0.025** | **1.63 (1.09, 2.44)** | **0.018** |
| Overall overweight×Stress | 0.79 (0.46, 1.34) | 0.380 | 0.62 (0.20, 1.91) | 0.410 | 0.80 (0.55, 1.15) | 0.230 |
| **Model d** |  |  |  |  |  |  |
| Abdominal thinness | **1.65 (1.05, 2.61)** | **0.031** | 1.47 (0.68, 3.16) | 0.324 | 1.31 (0.89, 1.93) | 0.167 |
| Abdominal overweight | 1.16 (0.76, 1.77) | 0.500 | 1.16 (0.78, 1.74) | 0.458 | 0.91 (0.67, 1.23) | 0.532 |
| Depression | **3.78 (1.39, 10.24)** | **0.009** | **3.18 (1.07, 9.40)** | **0.037** | **2.51 (1.31, 4.80)** | **0.005** |
| Abdominal thinness×Depression | **0.54 (0.33, 0.88)** | **0.014** | **0.53 (0.30, 0.92)** | **0.024** | 0.99 (0.63, 1.55) | 0.971 |
| Abdominal overweight×Depression | 1.55 (0.89, 2.69) | 0.121 | 1.57 (0.87, 2.83) | 0.132 | **2.41 (1.58, 3.68)** | **< 0.001** |
| **Model e** |  |  |  |  |  |  |
| Abdominal thinness | 1.61 (0.92, 2.82) | 0.094 | 1.82 (0.79, 4.17) | 0.159 | **1.49 (1.12, 1.98)** | **0.006** |
| Abdominal overweight | 1.23 (0.76, 2.00) | 0.408 | 1.22 (0.99, 1.50) | 0.068 | 1.06 (0.57, 1.98) | 0.850 |
| Anxiety | **2.84 (1.55, 5.19)** | **0.001** | **3.52 (1.99, 6.22)** | **< 0.001** | **2.42 (1.27, 4.61)** | **0.007** |
| Abdominal thinness×Anxiety | 0.74 (0.36, 1.54) | 0.423 | 0.53 (0.20, 1.44) | 0.214 | 0.81 (0.45, 1.46) | 0.485 |
| Abdominal overweight×Anxiety | 1.29 (0.57, 2.90) | 0.544 | 1.29 (0.56, 3.00) | 0.551 | 1.46 (0.64, 3.31) | 0.366 |
| **Model f** |  |  |  |  |  |  |
| Abdominal thinness | 1.24 (0.77, 1.98) | 0.377 | 1.03 (0.46, 2.30) | 0.950 | 1.13 (0.79, 1.62) | 0.511 |
| Abdominal overweight | 1.60 (0.96, 2.68) | 0.073 | 1.57 (0.90, 2.73) | 0.116 | **1.48 (1.15, 1.91)** | **0.003** |
| Stress | 1.76 (0.78, 3.98) | 0.171 | 1.20 (0.64, 2.24) | 0.572 | 1.32 (0.81, 2.16) | 0.261 |
| Abdominal thinness×Stress | 1.16 (0.58, 2.32) | 0.669 | 1.62 (0.87, 2.99) | 0.128 | **1.64 (1.13, 2.39)** | **0.010** |
| Abdominal overweight×Stress | 0.84 (0.48, 1.46) | 0.528 | 1.00 (0.58, 1.71) | 0.989 | 0.92 (0.64, 1.33) | 0.656 |

Abbreviations: BRI, the Behavioral Regulation Index; GEC, the Global Executive Composite; MI, the Metacognition Index.

^1^ All models (a to f) were adjusted for social-demographic factors (i.e. age, sex, parental highest education, and family gross income) and individual lifestyle behaviors (i.e. screen exposure, night sleep duration, and physical activity).

The bold words represent the P values less than 0.05.

**Table S16.** Moderating effects of depression in the association between abdominal weight spectrum and executive function problems in adolescents^1^.

|  | The Global Executive Composite (GEC) | | | | | | | | |
| --- | --- | --- | --- | --- | --- | --- | --- | --- | --- |
|  | The Behavioral Regulation Index (BRI) | | |  | The Metacognition Index (MI) | | | | |
|  | Inhibit | Emotion control | Shift |  | Working memory | Plan | Initiate | Organize | Monitor |
| Abdominal thinness | 1.24 (0.74, 2.06) | 1.66 (0.71, 3.88) | 1.52 (0.68, 3.39) |  | 1.28 (0.90, 1.82) | **1.45 (1.09, 1.92)*** | 1.43 (0.81, 2.55) | 1.27 (0.92, 1.75) | 1.17 (0.80, 1.70) |
| Abdominal overweight | 1.40 (0.88, 2.24) | 1.31 (0.88, 1.94) | 1.50 (0.93, 2.40) |  | 0.96 (0.74, 1.24) | 1.05 (0.55, 1.98) | 0.84 (0.41, 1.73) | 1.07 (0.69, 1.66) | 0.95 (0.77, 1.17) |
| Depression | 2.27 (0.86, 6.01) | **3.43 (1.31, 9.02)*** | **3.16 (1.64, 6.07)**** |  | **2.77 (1.41, 5.44)**** | **1.96 (1.28, 3.00)**** | **1.82 (1.02, 3.22)*** | 1.89 (0.65, 5.45) | **1.96 (1.33, 2.88)**** |
| Abdominal thinness×Depression | 0.76 (0.40, 1.45) | **0.44 (0.21, 0.91)*** | 0.59 (0.21, 1.63) |  | 0.74 (0.45, 1.20) | 0.99 (0.78, 1.25) | 0.93 (0.51, 1.68) | 1.12 (0.38, 3.34) | 1.28 (0.59, 2.76) |
| Abdominal overweight×Depression | 1.34 (0.62, 2.90) | 1.32 (0.74, 2.35) | 1.21 (0.52, 2.82) |  | **1.65 (1.06, 2.55)*** | 1.25 (0.46, 3.38) | **3.08 (1.24, 7.63)*** | 0.87 (0.08, 9.94) | 1.31 (0.66, 2.58) |

^1^ Adjusted for social-demographic factors (i.e. age, sex, parental highest education, and family gross income) and individual lifestyle behaviors (i.e. screen exposure, night sleep duration, and physical activity).

The bold words represent the P values less than 0.05.

* *P* < 0.05, ** *P* < 0.01, *** *P* < 0.001.

**Table S17.** Simple effects of abdominal weight spectrum on executive function problems stratified by depression condition in adolescents^1^.

|  | GEC problem | | BRI problem | | MI problem | |
| --- | --- | --- | --- | --- | --- | --- |
|  | OR (95% CI) | P value | OR (95% CI) | P value | OR (95% CI) | P value |
| **No depression** |  |  |  |  |  |  |
| Abdominal weight spectrum |  |  |  |  |  |  |
| Normal | Ref. |  | Ref. |  | Ref. |  |
| Thinness | **1.61 (1.08, 2.38)** | **0.019** | 1.43 (0.68, 3.01) | 0.341 | 1.31 (0.94, 1.83) | 0.108 |
| Overweight | 1.13 (0.71, 1.79) | 0.610 | 1.12 (0.68, 1.84) | 0.647 | 0.89 (0.66, 1.20) | 0.445 |
| **Depression** |  |  |  |  |  |  |
| Abdominal weight spectrum |  |  |  |  |  |  |
| Normal | Ref. |  | Ref. |  | Ref. |  |
| Thinness | 0.85 (0.42, 1.72) | 0.657 | 0.78 (0.30, 2.03) | 0.604 | 1.26 (0.76, 2.10) | 0.371 |
| Overweight | **1.80 (1.22, 2.65)** | **0.003** | **2.00 (1.25, 3.20)** | **0.004** | **2.26 (1.69, 3.01)** | **< 0.001** |

Abbreviations: BRI, the Behavioral Regulation Index; GEC, the Global Executive Composite; MI, the Metacognition Index.

^1^ Adjusted for social-demographic factors (i.e. age, sex, parental highest education, and family gross income) and individual lifestyle behaviors (i.e. screen exposure, night sleep duration, and physical activity).

The bold words represent the P values less than 0.05.

**Table S18.** Simple effects of abdominal weight spectrum on executive function problems stratified by depression condition in adolescents^1^.

|  | The Global Executive Composite (GEC) | | | | | | | | |
| --- | --- | --- | --- | --- | --- | --- | --- | --- | --- |
|  | The Behavioral Regulation Index (BRI) | | |  | The Metacognition Index (MI) | | | | |
|  | Inhibit | Emotion control | Shift |  | Working memory | Plan | Initiate | Organize | Monitor |
| **No depression** |  |  |  |  |  |  |  |  |  |
| Abdominal weight spectrum |  |  |  |  |  |  |  |  |  |
| Normal | Ref. | Ref. | Ref. |  | Ref. | Ref. | Ref. | Ref. | Ref. |
| Thinness | 1.21 (0.73, 2.01) | 1.63 (0.73, 3.68) | 1.51 (0.72, 3.19) |  | 1.27 (0.92, 1.77) | **1.46 (1.09, 1.96)*** | 1.43 (0.81, 2.52) | 1.33 (0.98, 1.81) | 1.15 (0.79, 1.69) |
| Overweight | 1.37 (0.81, 2.31) | 1.25 (0.75, 2.07) | 1.42 (0.83, 2.43) |  | 0.93 (0.73, 1.19) | 1.06 (0.57, 1.96) | 0.82 (0.40, 1.67) | 1.06 (0.62, 1.82) | 0.93 (0.77, 1.14) |
| **Depression** |  |  |  |  |  |  |  |  |  |
| Abdominal weight spectrum |  |  |  |  |  |  |  |  |  |
| Normal | Ref. | Ref. | Ref. |  | Ref. | Ref. | Ref. | Ref. | Ref. |
| Thinness | 0.93 (0.41, 2.11) | 0.71 (0.23, 2.19) | 0.86 (0.27, 2.75) |  | 0.93 (0.40, 2.16) | 1.38 (0.85, 2.24) | 1.30 (0.69, 2.46) | 1.25 (0.43, 3.67) | 1.43 (0.91, 2.24) |
| Overweight | **1.98 (1.29, 3.03)**** | **1.90 (1.29, 2.80)**** | 1.97 (1.00, 3.89) |  | **1.68 (1.24, 2.28)**** | 1.29 (0.76, 2.21) | **2.81 (1.67, 4.74)***** | 1.00 (0.09, 11.65) | 1.27 (0.67, 2.38) |

^1^ Adjusted for social-demographic factors (i.e. age, sex, parental highest education, and family gross income) and individual lifestyle behaviors (i.e. screen exposure, night sleep duration, and physical activity).

The bold words represent the P values less than 0.05.

* *P* < 0.05, ** *P* < 0.01, *** *P* < 0.001.
